# The Devil Is in the Details: Incomplete Reporting in Preclinical Animal Research

**DOI:** 10.1371/journal.pone.0166733

**Published:** 2016-11-17

**Authors:** Marc T. Avey, David Moher, Katrina J. Sullivan, Dean Fergusson, Gilly Griffin, Jeremy M. Grimshaw, Brian Hutton, Manoj M. Lalu, Malcolm Macleod, John Marshall, Shirley H. J. Mei, Michael Rudnicki, Duncan J. Stewart, Alexis F. Turgeon, Lauralyn McIntyre, Canadian Critical Care Translational Biology Group

**Affiliations:** 1 Clinical Epidemiology Program, The Ottawa Hospital Research Institute, Ottawa, Ontario, Canada; 2 Faculty of Medicine, University of Ottawa, Ottawa, Ontario, Canada; 3 School of Epidemiology Public Health and Preventive Medicine, University of Ottawa, Ottawa, Ontario, Canada; 4 Department of Medicine, University of Ottawa, Ottawa, Ontario, Canada; 5 Division of Clinical Neurosciences, University of Edinburgh, Edinburgh, United Kingdom; 6 Department of Surgery (Critical Care), University of Toronto, Toronto, Ontario, Canada; 7 Regenerative Medicine Program, The Ottawa Hospital Research Institute, Ottawa, Ontario, Canada; 8 Department of Cell and Molecular Medicine, University of Ottawa, Ottawa, Ontario, Canada; 9 Population Health and Optimal Health Practices Unit (Trauma – Emergency – Critical Care Medicine), Centre de Recherche du CHU de Québec (Enfant-JésusHospital), Université Laval, Québec City, Québec, Canada; 10 Division of Critical Care Medicine, Department of Anesthesiology, Université Laval, Québec City, Québec, Canada; 11 Department of Medicine (Division of Critical Care), University of Ottawa, Ottawa, Ontario, Canada; Central South University, CHINA

## Abstract

Incomplete reporting of study methods and results has become a focal point for failures in the reproducibility and translation of findings from preclinical research. Here we demonstrate that incomplete reporting of preclinical research is not limited to a few elements of research design, but rather is a broader problem that extends to the reporting of the methods and results. We evaluated 47 preclinical research studies from a systematic review of acute lung injury that use mesenchymal stem cells (MSCs) as a treatment. We operationalized the ARRIVE (Animal Research: Reporting of In Vivo Experiments) reporting guidelines for pre-clinical studies into 109 discrete reporting sub-items and extracted 5,123 data elements. Overall, studies reported less than half (47%) of all sub-items (median 51 items; range 37–64). Across all studies, the Methods Section reported less than half (45%) and the Results Section reported less than a third (29%). There was no association between journal impact factor and completeness of reporting, which suggests that incomplete reporting of preclinical research occurs across all journals regardless of their perceived prestige. Incomplete reporting of methods and results will impede attempts to replicate research findings and maximize the value of preclinical studies.

## Introduction

Completeness of reporting in clinical trials has improved in journals that endorse reporting guidelines [1–3]. Although preclinical reporting guidelines are relatively recent, publishers, journals, funders, and scientific societies are embracing their use with the intent of improving the completeness of reporting [4]. The ARRIVE guidelines [5] is the most widely endorsed [4] preclinical reporting guidance. These guidelines were developed in response to evidence that a failure to report research methods and results appropriately is a widespread problem in biomedical research [6]. The National Institutes of Health (NIH) has also developed guidance for both authors and publishers [7]; and publishers such as the Nature Publishing Group have implemented journal level checklists [8]. Ultimately, improved reporting is part of a larger effort to improve the reproducibility and translation of preclinical research [9,10]. Complete reporting of methods and results will allow reviewers and readers to evaluate the experimental design and make better judgements about rigor [11].

**Fig 1:**
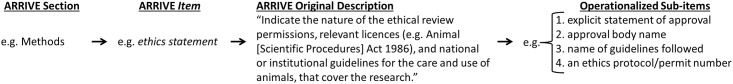
The ARRIVE guidelines have six Sections: Title, Abstract, Introduction, Methods, Results, Discussion. Each Section has at least one *item* (e.g. *ethics statement*) with a description which we operationalized into discrete yes/no sub-items.

Previous evaluations of preclinical reporting have focused on a limited scope of reporting such as blinding and randomization because failure to implement these methods is associated with exaggeration of efficacy in both clinical and preclinical studies [12–14]. Here we sought to determine the scope of reporting in greater detail by focusing on a particular preclinical animal model (acute lung injury) and treatment (MSCs) as part of a systematic review [15]. Our search strategy resulted in 5,391 total records which after screening left 47 English language articles that met our prespecified criteria from our protocol [16] (see methods and S1 Fig, S1 Table). We used the ARRIVE guidelines to assess reporting because it is the most widely recognized and detailed guideline for preclinical studies, endorsed by more than 600 biomedical journals [4]. We assessed 109 individual sub-items scored as yes/no for reporting. These sub-items were nested across 17 broader ARRIVE *items* (herein italicised) from the six Sections (herein capitalized) of the ARRIVE guidelines. For example, within the Section ‘Methods’, the *item* ‘*ethical statement*’ was operationalized into four sub-items: 1) explicit statement of approval; 2) approval body name; 3) name of guidelines followed; and 4) an ethics protocol/permit number (Fig 1). For a complete list of all ARRIVE Sections, *items*, and sub-items see S2 Table.

## Results

**Fig 2:**
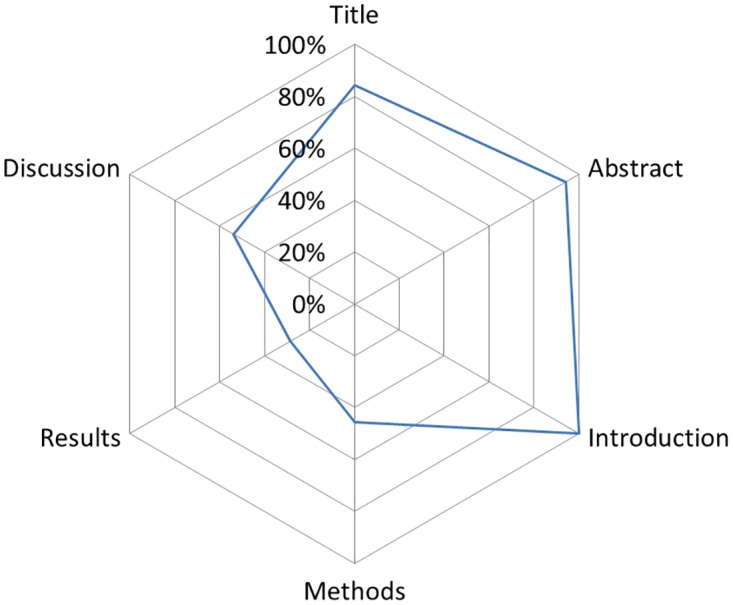
The six ARRIVE Sections are listed around the circumference of the chart starting with Title at twelve o’clock. The line represents the percentage of sub-items (e.g. species) reported for all studies per Section (e.g. Methods). For example, for the Section Title (84%) we summed the total number of reported ‘yes’ sub-items (119) and then divided it by the number of independent sub-items (3) multiplied by the total number of studies (47): 119/(3*47) = 0.84.

**Fig 3:**
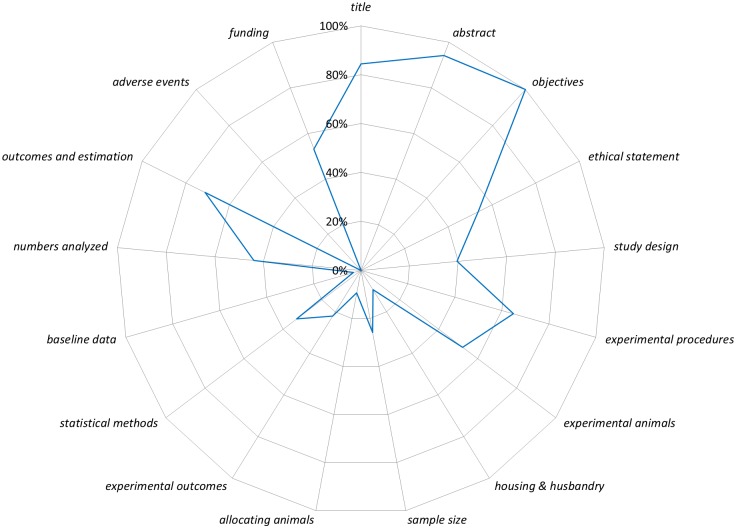
The 17 ARRIVE *items* are listed around the circumference of the chart starting with *title* at twelve o’clock. The line represents the percentage of sub-items (e.g. species) reported for all studies per *item* (e.g. *experimental animals*). For example, for the *item title* (84%) we summed the total number of reported ‘yes’ sub-items (119) and then divided it by the number of independent sub-items (3) multiplied by the total number of studies (47): 119/(3*47) = 0.84.

Fig 2 presents a graphical summary of the completeness of reporting for all sub-items aggregated into the six Sections of the ARRIVE guidelines. Reporting for the Title, Abstract, and Introduction Sections were generally high (84% to 100% of sub-items per section), whereas Methods, Results, and Discussion Sections were generally lower (26% to 54% of sub-items per section; Fig 2). Fig 3 presents a graphical summary of the completeness of reporting for all sub-items aggregated into the 17 *items* of the ARRIVE guidelines. Reporting of *items* from the Methods Section ranged from 9% (*allocating animals to experimental groups*, *housing and husbandry*) to 65% (*experimental procedures;* S2–S9 Figs). Reporting of *items* from the Results Section ranged from 0% (*adverse events*) to 71% (*outcomes and estimation*; S10 Fig). For the Introduction and Discussion Sections, the *items* were generally well reported (range 84%–100%) except for *funding* (53%) which had two of four sub-items poorly reported (role of funders described, 2%; statement of competing/conflict of interest, 57%; S11 Fig). Exact values for all 109 sub-items are in S3 Table.

**Fig 4:**
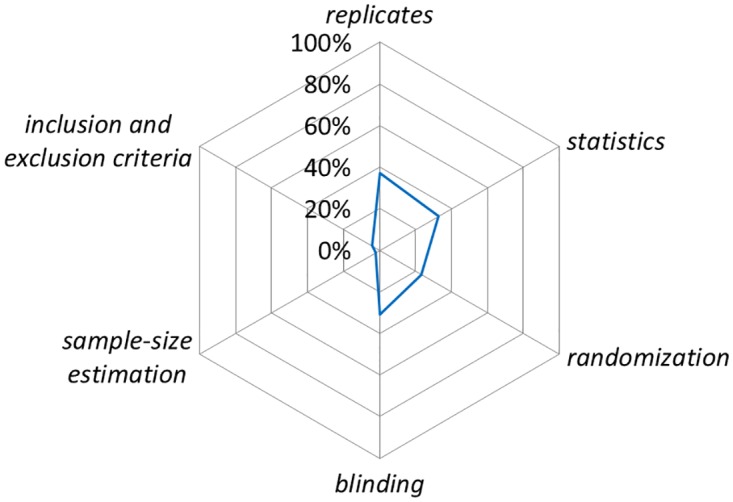
The six NIH ‘core’ reporting *items* are listed around the circumference of the chart starting with *replicates* at twelve o’clock. The line represents the percentage of ARRIVE sub-items (e.g. was a sample size calculation conducted) reported ‘yes’ for all studies that matched with each NIH core *item*. ARRIVE sub-items matched with NIH *items* are listed in S4 Table.

**Fig 5:**
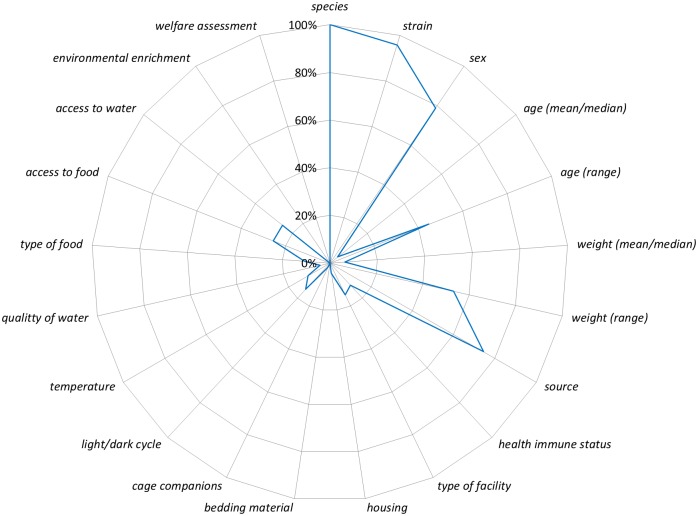
The ARRIVE sub-items that aligned with the NIH’s biological materials: animals reporting recommendation are listed around the circumference of the chart starting with species at twelve o’clock. The line represents the percentage of 47 studies that reported the sub-item (e.g. all 47 studies reported the sub-item species).

**Fig 6:**
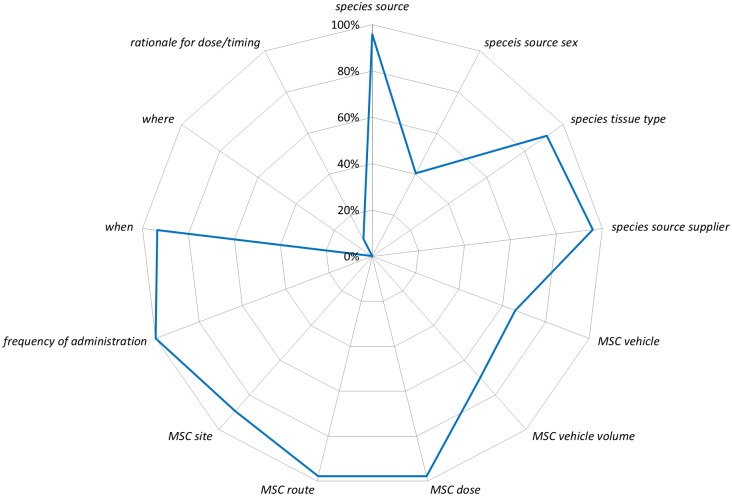
The ARRIVE sub-items that aligned with the NIH’s biological materials: cell lines reporting recommendation are listed around the circumference of the chart starting with species source at twelve o’clock. The line represents the percentage of 47 studies that reported the sub-item (e.g. 96% of studies reported the sub-item species source).

Given the large number of sub-items from the ARRIVE guidelines we focused our analysis on a more narrow range of sub-items using the National Institutes of Health (NIH) Principles and Guidelines for Reporting Preclinical Research [17]. The NIH includes a ‘core’ set of reporting *items* (*replicates*, *statistics*, *randomization*, *blinding*, *sample-size estimation*, *inclusion and exclusion criteria*) adopted from Landis and colleagues [18]. To assess these ‘core’ reporting *items* we created composites of sub-items from the ARRIVE guidelines. None of the NIH core *items* were reported more than 40% of the time (Fig 4), and the elements of both *sample-size estimation* and *inclusion/exclusion criteria* were rarely reported (2% and 4%, respectively). Although our composite item for randomization was reported 23% of the time (Fig 4; S4 Table), the use of term randomization was reported in 22 of 47 studies (47%) whereas random sequence generation was never reported. The NIH also recommends best practices for the description of biological materials for *animals* and *cells*. Biological materials for *animals* aligns with the *experimental animals* and *housing and husbandry items* from the ARRIVE guidelines. Reporting for sub-items for these two *items* ranged from 0% (bedding material, environmental enrichment, welfare assessment) to 100% (species; Fig 5). Biological materials *cells* align with *experimental procedures* for MSC administration (which was coded separately from acute lung injury inducement and control). Reporting of sub-items from this *item* ranged from 0% (where [i.e. home cage]) to 98% (MSC dose, MSC route of administration; Fig 6).

Despite the limitations of journal impact factor [19,20], it is widely used to assess the quality and prestige of journals, articles, and even scientists. We assessed whether journal impact factor (2013) [21] was associated with completeness of reporting by dividing the 47 studies into low (impact factor < = 4, n = 28) and high (impact factor >4, n = 19) groups. We found no difference in the percentage for completeness of reporting between the low (median 51 items, min = 38, max = 64) and high impact factor journals (median 49 items, min = 37, max = 61; p = 0.66) using a Mann-Whitney test. We also found no difference in the percentage for completeness of reporting using our second approach between low (median 50 items, min = 38, max = 63), mid (median 51 items; min = 43, max = 64) and high (median 52 items, min = 37, max = 61; p = 0.91). We suggest that this indicates that poor reporting of details is a community wide problem that is not specific to individual laboratories, journals, or publishers. Since no individual study (or journal) reported an exceptional number of sub-items (S12 Fig) in our sample there are no examples of how to implement good reporting practice. Our results also suggest that impact factor should not be used as a surrogate indicator for better reported research [20].

A growing number of journals and publishers are endorsing both the ARRIVE [4] and NIH guidelines [17]. However, there is little evidence that they are being implemented during the drafting and peer review of manuscripts [22–24]. In our sample of 47 papers from 38 unique journals there were only nine journals (24%) that mentioned the ARRIVE guidelines anywhere on their website (March 2014). The ARRIVE guidelines were developed with the specific aim of improving the completeness of reporting for preclinical studies [5,6]. To assess whether the publication of the ARRIVE guidelines were associated with the completeness of reporting, we allowed for a one-year time period post-ARRIVE publication and compared the median number of items reported before and after. We found a statistically significant increase in the median number of sub-items reported in studies that were published after the ARRIVE guidelines (median before 49, min = 37, max = 64; median after 53.5, min = 38, max = 63; p = 0.02) using a Mann-Whitney test. However, the absolute difference in medians was quite small (less than 5 items) and the post-ARRIVE group still reported less than 50% of the assessed reporting items S13 Fig. We show here a small difference in studies published after the ARRIVE guidelines were introduced which may signal that journals are shifting from endorsement to implementation. However, there is no apparent pattern S13 Fig to which sub-items are being reported more frequently suggesting this may be driven by extraneous factors not related to the early adoption of the ARRIVE guidelines by authors and journals [20].

## Discussion

Although many of the *items*, such as *housing and husbandry*, that we assessed in the ARRIVE guidelines are not NIH ‘core’ *items*, there is growing recognition for their importance since these characteristics may impact the health of the animals and in turn lead to varied treatment responses [25]. Documenting these details is important to address potential reasons for discrepancies between results from different labs (i.e. replication) within the same preclinical animal models [26]; facilitate replication of methods; and to ensure that the maximum value of primary studies is realized in syntheses such as meta-analyses. We also found that less controversial reporting sub-items were missing. For instance, the numbers of experimental groups were often inconsistent between the methods and results sections of the studies (70% of studies matched). This indicates that neither authors, nor reviewers are closely following the experimental design or results sections; this may increase the risk of biased results

Low levels of complete reporting relative to the ARRIVE guidelines has been found previously [24] although more limited in scope than the current assessment. We show that reporting of details is generally incomplete whether they are a handful of core *items* or the more comprehensive ARRIVE *items*. The incomplete reporting of these details directly impedes the ability to assess the validity of the experiments. Many of the sub-items we assessed relate to the internal validity or rigor of these experiments (e.g. blinding and randomization) [11,27]. Appropriate implementation and reporting of these measures would reduce the risk that estimates of efficacy will be biased from systematic variation and provide greater confidence that causal variables have been identified [12–14]. These preclinical experiments of efficacy use a disease construct (ALI) that models the human condition acute respiratory distress. Here again, clearly reporting on sub-items related to the construct validity directly informs readers about the translatability of the findings from the preclinical model to the human health condition [11,27], but even basic information like sex, age, and timing of MSC administration were routinely not reported. None of the sub-items directly assessed the external validity or generalizability (i.e. same or similar results in multiple models or multiple labs) [11,27] nor did they assess directly assess the replicability or reproducibility of the included studies. We suggest that these concepts can be best assessed through preclinical systematic reviews of in vivo animal studies [28].

## Materials and Methods

### Selection of Studies

We utilized 47 English language studies there were previously identified in a systematic review of acute lung injury and MSCs [15]. A detailed protocol for the systematic was pre-registered [29] and published [16] prior to conducting the research. A list of the included studies is included in S1 Table and general characteristics of the included studies are published in our systematic review [15].

### Operationalization of ARRIVE Guidelines

The ARRIVE guidelines consist of six Sections (Title, Abstract, Introduction, Methods, Results, Discussion) and 20 *items* each of which contains recommendations of multiple, independent concepts to be evaluated. We adopted the language used by the ARRIVE guidelines such that we refer to the highest level as a ‘Section’ (e.g. Methods; herein capitalized) and within a Section there are *items* (e.g. *experimental procedures*; herein italicised), and within *items* there are recommendations. We divided the recommendations into components and evaluated which were relevant to acute lung injury and which were not. For the recommendation components that were relevant we then operationalized them into sub-items (e.g. drug dose; drug volume, etc.; herein lowercase non-italicised) by referring to the original ARRIVE Guidelines publication, examples provided by the N3CRs, and consulting with our preclinical and clinical experts in acute lung injury/acute respiratory distress. Our approach is similar to Moberg-Mogren and Nelson’s rules for sub-items used with the CONSORT instrument [30]. To adapt the ARRIVE guidelines to the reporting of preclinical studies of acute lung injury, we followed four basic steps:

**Step 1:** Our review focused on the reporting of the Methods and Results Sections of the studies because information in these Sections is crucial for an evaluation of the scientific validity of the study. We also included the Title, Abstract, Sections, as they are fundamental to identifying relevant studies, as well as the *objectives* and *funding items* from the Introduction and Discussion Sections in our reporting evaluation. Thus, we retained 17 *items* (1, 2, 4–17, and 20) and removed *item* 3 (*background*) and items 18–19 (*interpretation/scientific implications; generalisability/translation*) from the original ARRIVE guideline.
**Step 2:** All of the recommendations for each of the retained *items* were divided into ‘recommendation components’. These components each captured only one issue or concept of reporting. For example, the ARRIVE recommendation for the Title Section is: “Provide as accurate and concise a description of the content of the study as possible”. We divided this recommendation into two components: 1) an accurate description of the study; and 2) a concise description of the study. In total, we divided the recommendations into 91 components without adding or removing any recommendations for the 17 *items* included. We then removed components that were deemed irrelevant because they would not apply to pre-clinical acute lung injury (i.e. tank shape and tank material) leaving a total of 89 components.
**Step 3:** We operationalized the 89 recommendation components into 109 sub-items such that a reviewer could score them as “yes they were reported” or “no they were not reported” (see S2 Table). The sub-items also could only capture one issue or concept of reporting but they were operationalized such that they were relevant for acute lung injury models and objective to score. For example, the recommendation components for the Title Section (see Step 2 above) were operationalized into three sub-items (1. species studied; 2. disease modeled; 3. intervention tested) that captured the concepts ‘accurate’ and ‘concise’ in objective statements that could be rated as reported yes or no.
**Step 4:** We developed a framework for evaluating all 17 *items* and 109 sub-items. First, we evaluated the published study itself and any additional supplementary materials, but references to other studies were not evaluated. Second, sub-items evaluated in the study had to correspond to the same section as in the ARRIVE guidelines. Thus, if a sub-item was listed in the Methods Section of the ARRIVE guidelines, it was only evaluated if it appeared in the Methods Section of the study or the supplementary materials. The sub-items for funding could be located anywhere in the studies. Third, only the reporting of *in vivo* experiments to induce and test models of acute lung injury within the study were evaluated. Any other *in vivo*, *in vitro*, human, and other experiments were not evaluated. Fourth, for certain sub-items an algorithm was developed to follow that ensured each sub-item was scored in similar manner between studies. For example, since studies reported different numbers and types of outcomes we evaluated mortality as an outcome first and then the first reported outcome if mortality was not assessed in the study. Fifth, although the operationalized sub-items applied to studies almost universally since the sample consisted of closely related studies (i.e. acute lung injury models treated with MSCs); certain sub-items varied between studies such as procedures to induce acute lung injury (e.g. bleomycin vs cecal ligation and puncture). Thus for few specific sub-items (e.g. drug vehicle, vehicle volume, dose) alternate examples were generated specifically for these less common procedures such as cecal ligation and puncture. Sixth, if authors specifically stated that a sub-item was no performed (e.g. analgesic administration, or randomization) or an event did not occur (i.e. no adverse events) they would still be scored as ‘yes’ reported.

### Data Extraction and Analysis

Each study and supplementary materials were assessed independently by two reviewers (Avey, MT; and Sullivan, KJ). Any discrepancy in the extracted data between these two reviewers was resolved by discussion and consensus, and if consensus could not be reached, a third party (McIntyre, L) was consulted. The two reviewers discussed the reporting checklist for all sub-items to ensure there was agreement on the meaning of each sub-item, and then each reviewer independently piloted the checklist on three studies. Any discrepancies in interpretation between the reviewers was discussed and clarified.

Descriptive statistics were generated for all Sections, *items*, and sub-items of the ARRIVE guidelines (see S3 Table). For the NIH guidelines six core items, we assigned sub-items from ARRIVE that broadly matched the NIH’s descriptions (see S4 Table) and calculated descriptive statistics. For the comparison of low versus high impact factor journal publications and the association with completeness of reporting, we used the 2013 journal impact factor. We took two approaches: 1) we grouped them into low (<4; n = 28; min = 0, max = 3.65) and high (> = 4; n = 19; min = 4.02, max = 24.30); and 2) we group them into low (<3; n = 16, min = 0, max = 2.60), mid (3–5; n = 18; min = 3.05, max = 4.75), and high (>5, n = 13, min = 5.16, max = 24.30). For all 109 items, we treated them as having equal weight for this analysis, and if no impact factor could be found, we entered the impact factor as 0 (six studies). We analyzed the data with a Mann-Whitney test for the first approach and a Kruska-Wallis test for the second. For the comparison of studies published before versus after the ARRIVE guidelines were published (June 29^th^ 2010), we assumed a one year time lag in publication and assigned studies to groups based on their submission dates. If no submission date was available, then publication date was used (five studies). For all 109 items, again, we treated them as having equal weight for this analysis. As before, data was analyzed with a Mann-Whitney test. The unit of analysis for both the journal impact factor and before/after ARRIVE guidelines publication was the total number of reported items per study.

## Supporting Information

S1 Fig PRISMA Flow Diagram for identification, screening, eligibility, and included studies. (TIF)

S2 Fig The ARRIVE sub-items (e.g. ethics approval) from each of *ethical statement* and *study design* are listed around the *circumference* of the chart starting with the sub-item ethics approval at twelve o’clock. The line represents the percentage of 47 studies that reported the sub-item (e.g. 83% of studies reported the sub-item ethics approval).
(TIF)

S3 Fig The ARRIVE sub-items (e.g. drug or method) from *experimental procedures* (acute lung Injury model) are listed around the *circumference* of the chart starting with the sub-item drug or method at twelve o’clock. The line represents the percentage of 47 studies that reported the sub-item (e.g. 100% of studies reported the sub-item drug or method). *For methods of inducing acute lung injury that did not use a drug (i.e. cecal ligation and puncture) these sub-items were scored using alterative examples (see methods for details).
(TIF)

S4 Fig The ARRIVE sub-items (e.g. MSC species source) from *experimental procedures* (MSCs) are listed around the *circumference* of the chart starting with the sub-item MSC species source at twelve o’clock. The line represents the percentage of 47 studies that reported the sub-item (e.g. 96% of studies reported the sub-item MSC species source).
(TIF)

S5 Fig The ARRIVE sub-items (e.g. drug or method) from *experimental procedures* (control groups; and euthanasia) are listed around the *circumference* of the chart starting with the sub-item drug or method at twelve o’clock. The line represents the percentage of 47 studies that reported the sub-item (e.g. 96% of studies reported the sub-item drug or method.
(TIF)

S6 Fig The ARRIVE sub-items (e.g. species) from *experimental animals* are listed around the *circumference* of the chart starting with the sub-item species at twelve o’clock. The line represents the percentage of 47 studies that reported the sub-item (e.g. 100% of studies reported the sub-item species).
(TIF)

S7 Fig The ARRIVE sub-items (e.g. type of facility) from *housing and husbandry* are listed around the *circumference* of the chart starting with the sub-item type of facility at twelve o’clock. The line represents the percentage of 47 studies that reported the sub-item (e.g. 15% of studies reported the sub-item type of facility).
(TIF)

S8 Fig The ARRIVE sub-items (e.g. sample-size calculation) from each of *sample size* and *allocating animals to experimental groups* are listed around the *circumference* of the chart starting with the sub-item sample-calculation at twelve o’clock. The line represents the percentage of 47 studies that reported the sub-item (e.g. 2% of studies reported the sub-item sample-size calculation).
(TIF)

S9 Fig The ARRIVE sub-items (e.g. total # of outcomes listed in methods) from each of *experimental outcomes* and *statistical methods* are listed around the *circumference* of the chart starting with the sub-item total # of outcomes listed in methods at twelve o’clock. The line represents the percentage of 47 studies that reported the sub-item (e.g. 0% of studies reported the sub-item total # of outcomes listed in methods).
(TIF)

S10 Fig The ARRIVE sub-items (e.g. group weight (mean/median)) for the Results Section from each of *baseline data*, *numbers analysed*, *outcomes & estimation*, and *adverse events* are listed around the *circumference* of the chart starting with group weight (mean/median) at twelve o’clock. The line represents the percentage of 47 studies that reported the sub-item (e.g. 11% of studies reported the sub-item group weight (mean/median)).
(TIF)

S11 Fig The ARRIVE sub-items for the Title, Abstract, Introduction, and Discussion Sections are listed around the circumference of the chart starting with the sub-item *title*: species at twelve o’clock. The line represents the percentage of 47 studies that reported the sub-item (e.g. 57% of studies reported the sub-item *title*: species).
(TIF)

S12 Fig Heat map of reporting by *item* of the ARRIVE guidelines for studies submitted either before or after publication of ARRIVE based on a one year time lag from submission date. The total number of sub-items for each study was summed by *item* (e.g. *study design*) and divided by the total number of sub-items in that *item* (i.e for *title* there were three sub-items, thus each study could score: 0[none], 0.33[1 of 3], 0.67[2 of 3], or 1[3 of 3]). Colours were assigned with red = 0 (none reported), yellow = 0.5, green = 1 (all reported). Each column of colour represents one study (e.g. 2006 has two studies), single black lines separate years, and the double black line separates submitted before and after ARRIVE publication.
(TIF)

S13 Fig Heat map of reporting by *item* of the ARRIVE guidelines grouped by low and high impact factor studies. The total number of sub-items for each study was summed by *item* (e.g. *study design*) and divided by the total number of sub-items in that *item* (i.e for *title* there were three sub-items, thus each study could score: 0[none], 0.33[1 of 3], 0.67[2 of 3], or 1[3 of 3]). Colours were assigned with red = 0 (none reported), yellow = 0.5, green = 1 (all reported). Each column of colour represents one study and the double black line separates low impact factor (<4; n = 28; min = 0, max = 3.62) and high impact factor (> = 4; n = 19; min = 4.02, max = 24.03) study groups.
(TIF)

S1 Table References for Included Studies. (PDF)

S2 Table Operationalized Reporting sub-items from the ARRIVE Guidelines and Examples. (PDF)

S3 Table Number and Percentage of Studies Reporting for Each sub-item. (PDF)

S4 Table NIH Core Reporting *item* and Matched ARRIVE sub-items. (PDF)
