# Supplementary figures and images for: The Devil Is in the Details: Incomplete Reporting in Preclinical Animal Research
Source: PLoS One. 2016 Nov 17;11(11):e0166733. doi: 10.1371/journal.pone.0166733 (PMC5113978; doi:10.1371/journal.pone.0166733)

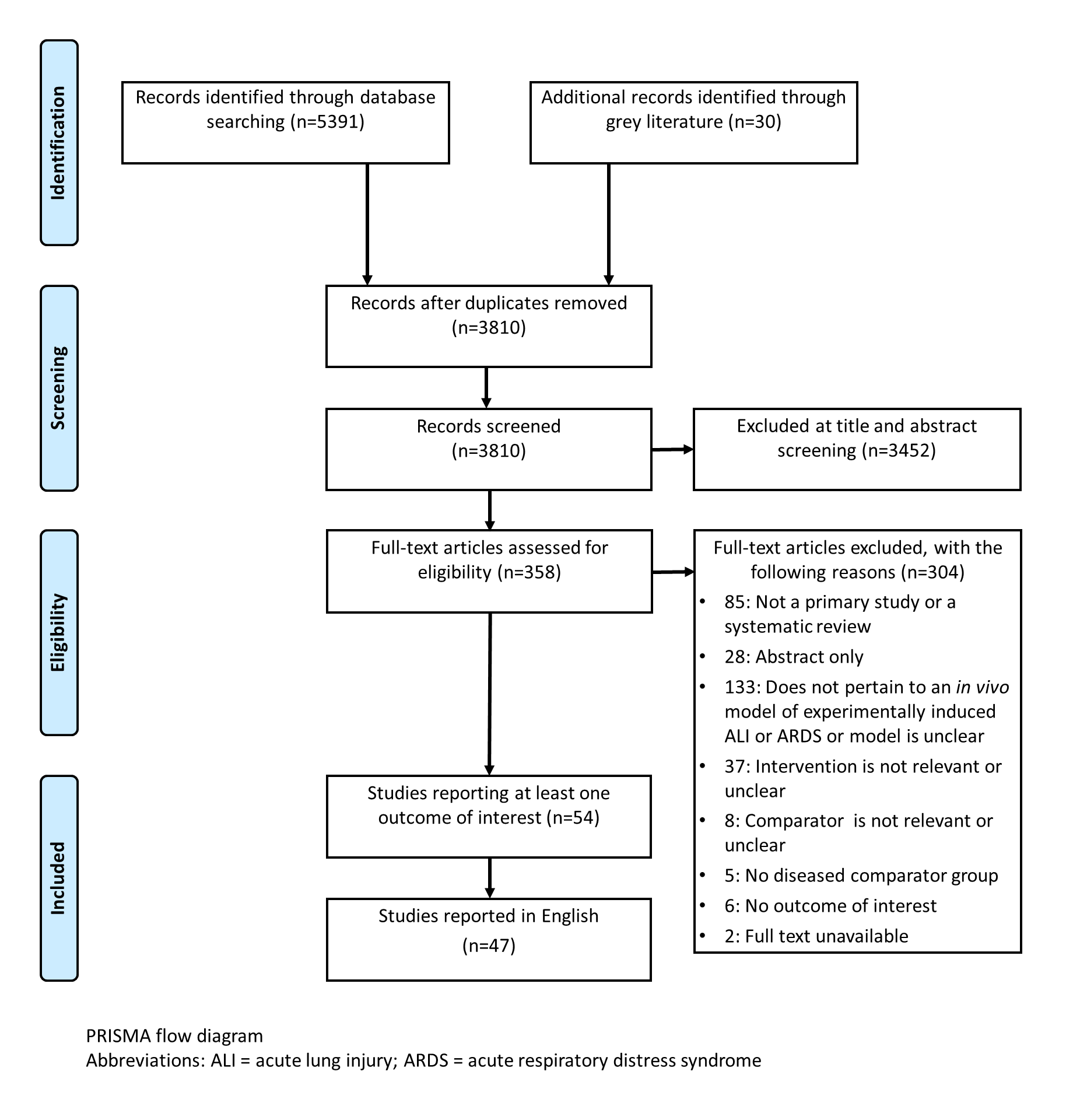

Supplement: S1 Fig — (TIF) [file pone.0166733.s001.tif]

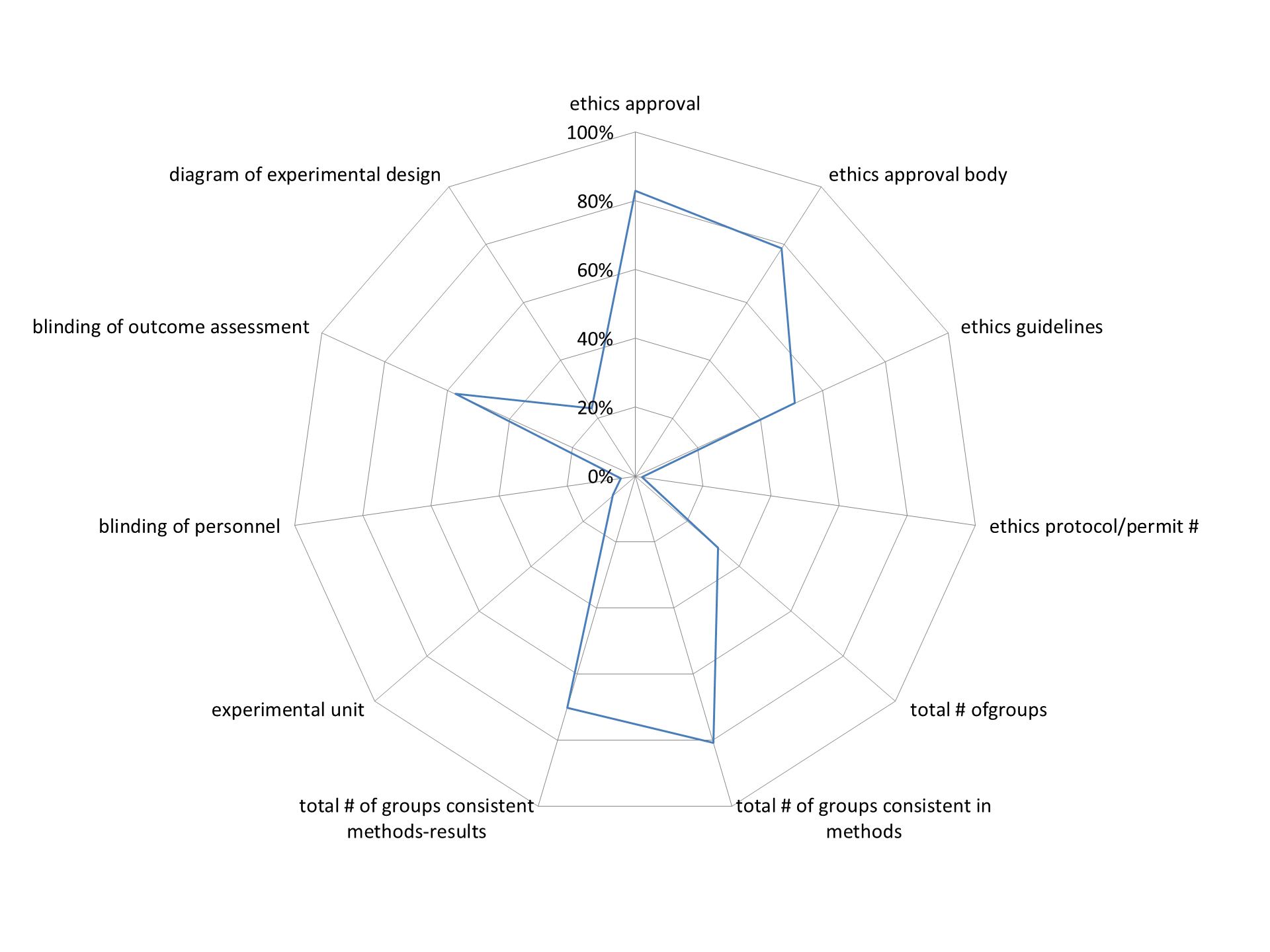

Supplement: S2 Fig — The line represents the percentage of 47 studies that reported the sub-item (e.g. 83% of studies reported the sub-item ethics approval). (TIF) [file pone.0166733.s002.tif]

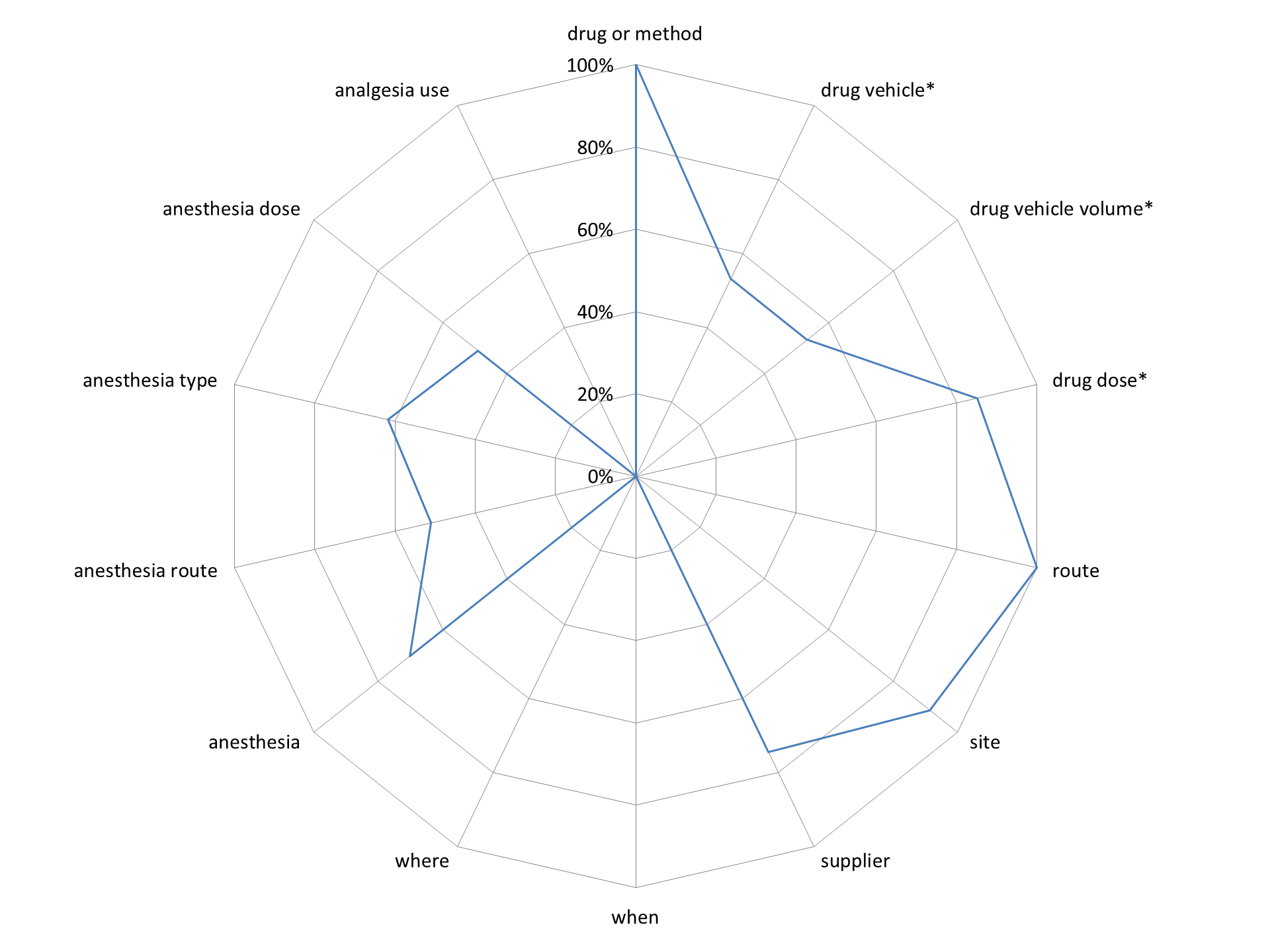

Supplement: S3 Fig — The line represents the percentage of 47 studies that reported the sub-item (e.g. 100% of studies reported the sub-item drug or method). *For methods of inducing acute lung injury that did not use a drug (i.e. cecal ligation and puncture) these sub-items were scored using alterative examples (see methods for details). (TIF) [file pone.0166733.s003.tif]

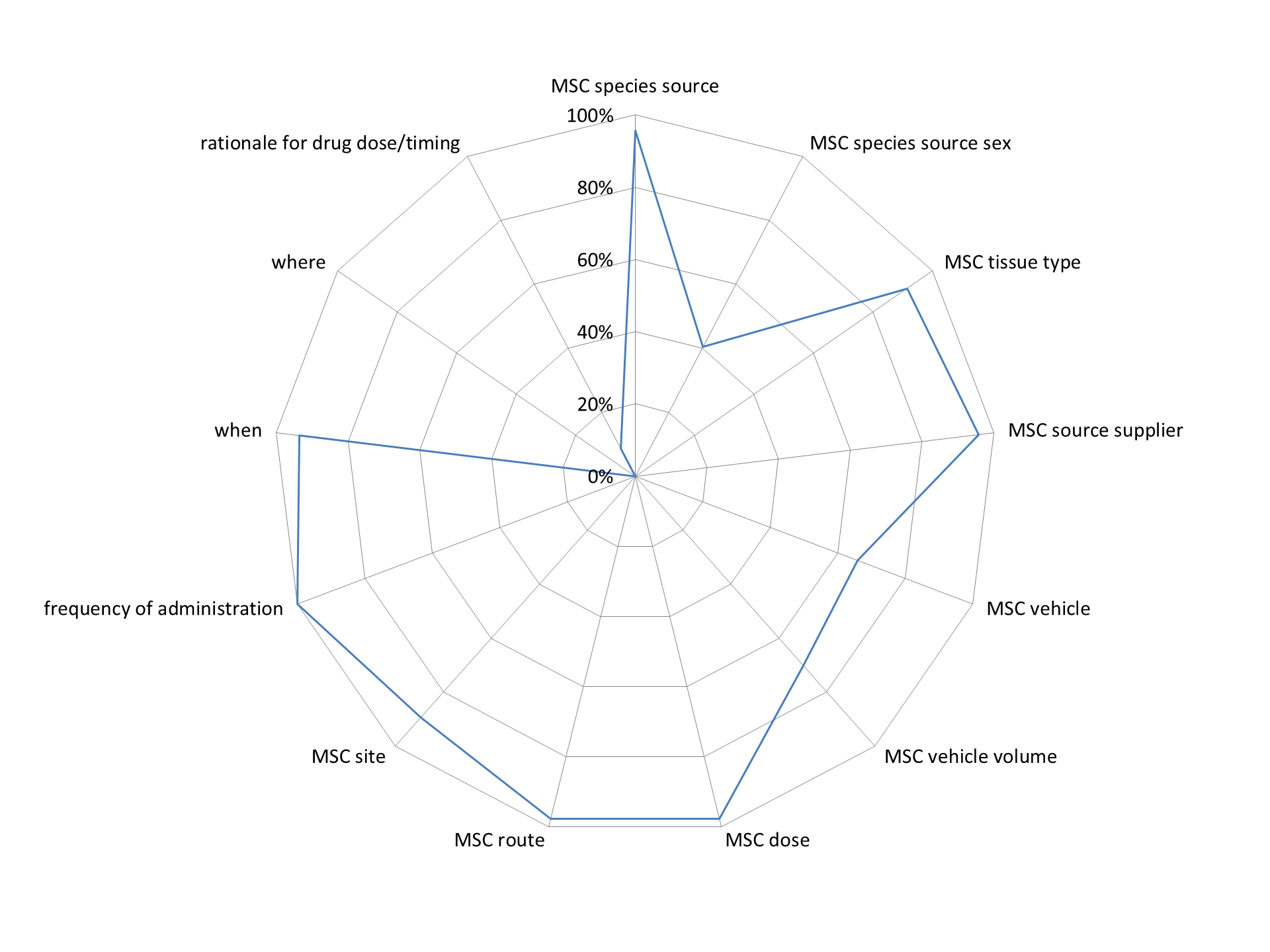

Supplement: S4 Fig — The line represents the percentage of 47 studies that reported the sub-item (e.g. 96% of studies reported the sub-item MSC species source). (TIF) [file pone.0166733.s004.tif]

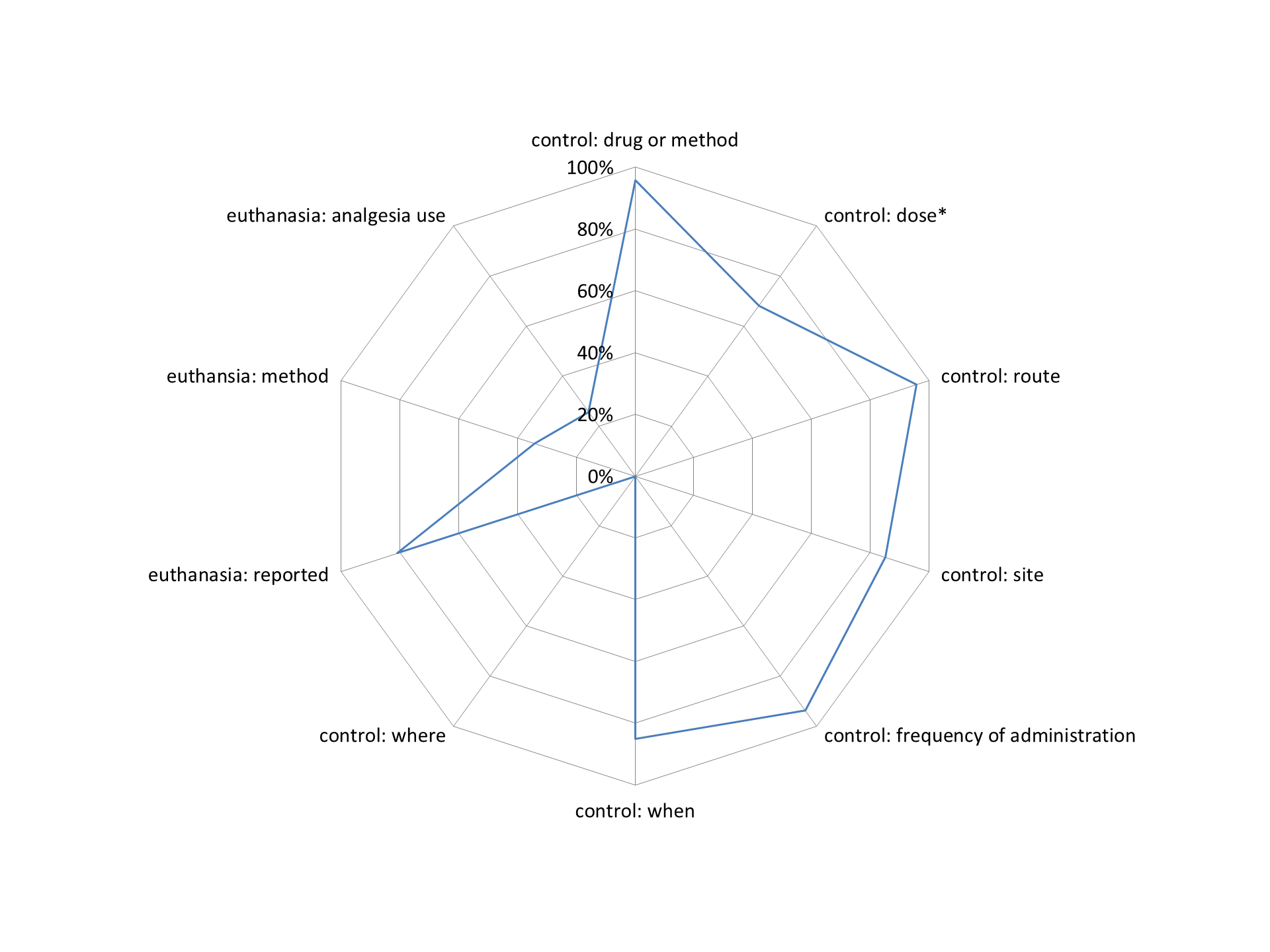

Supplement: S5 Fig — The line represents the percentage of 47 studies that reported the sub-item (e.g. 96% of studies reported the sub-item drug or method. (TIF) [file pone.0166733.s005.tif]

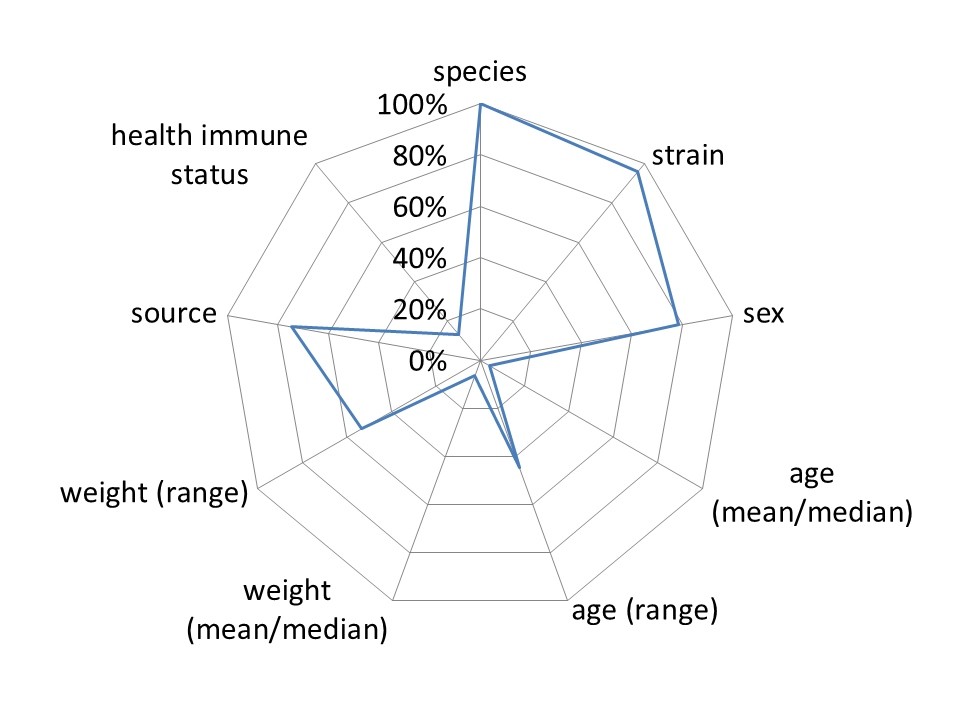

Supplement: S6 Fig — The line represents the percentage of 47 studies that reported the sub-item (e.g. 100% of studies reported the sub-item species). (TIF) [file pone.0166733.s006.tif]

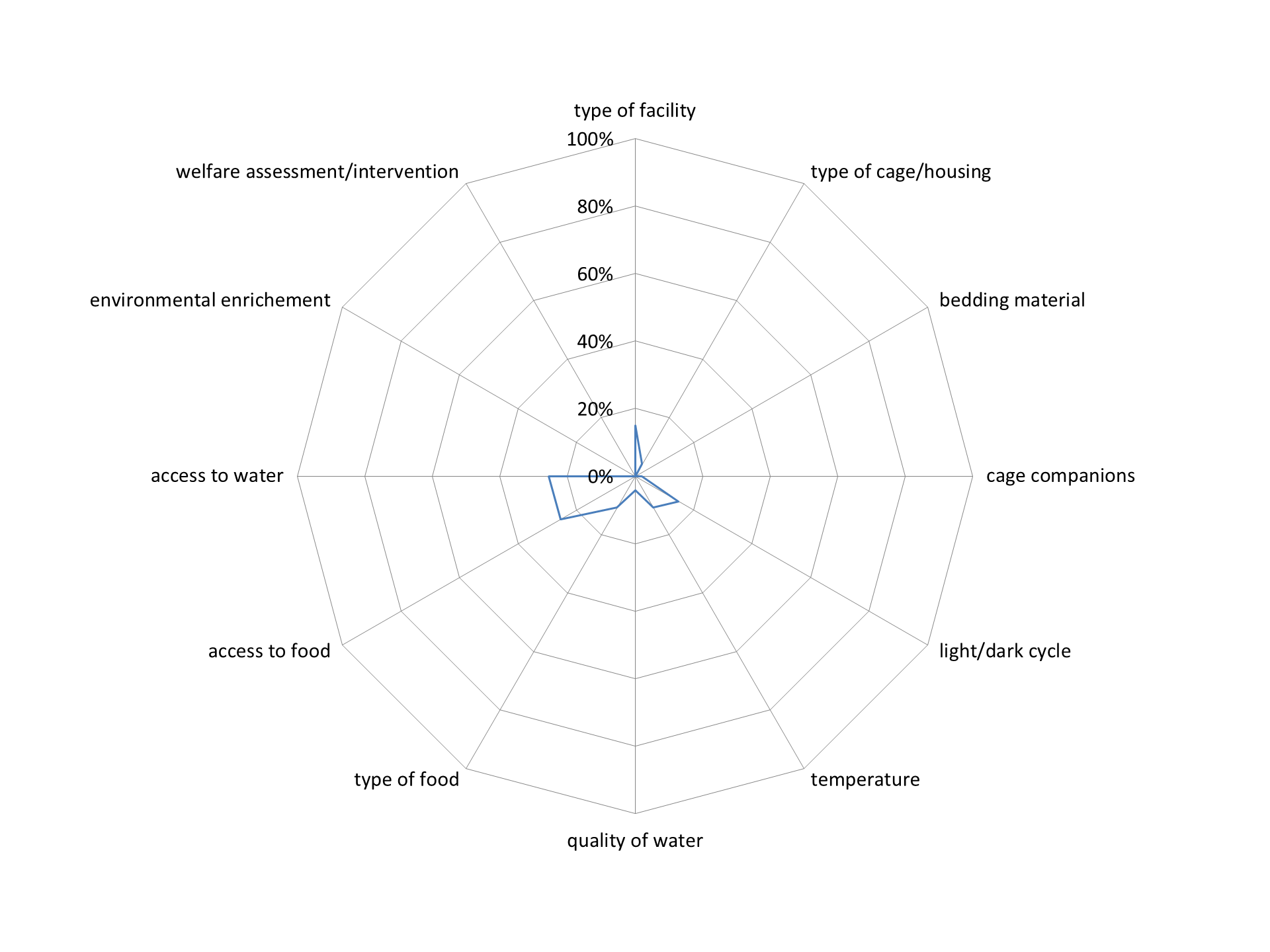

Supplement: S7 Fig — The line represents the percentage of 47 studies that reported the sub-item (e.g. 15% of studies reported the sub-item type of facility). (TIF) [file pone.0166733.s007.tif]

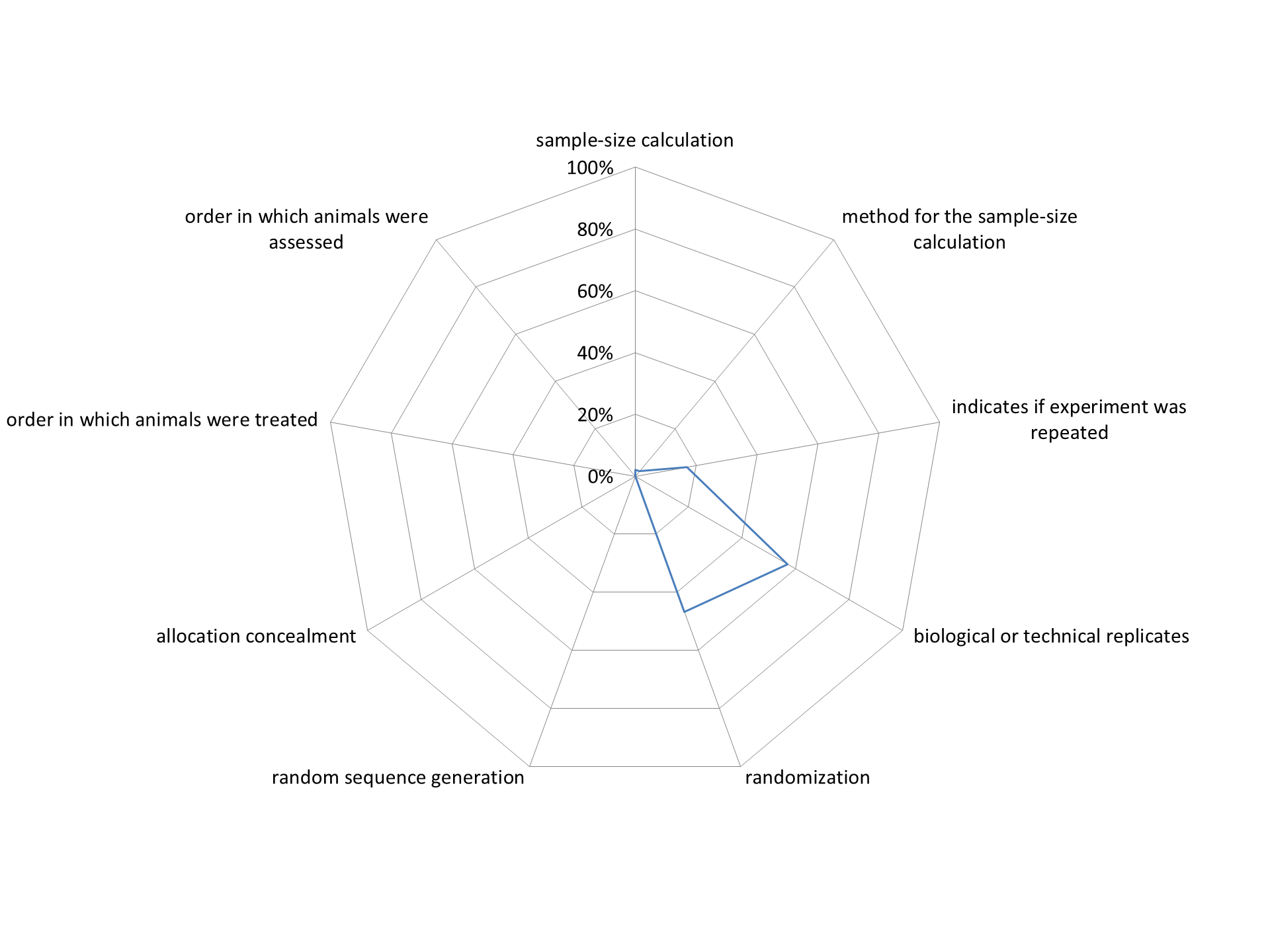

Supplement: S8 Fig — The line represents the percentage of 47 studies that reported the sub-item (e.g. 2% of studies reported the sub-item sample-size calculation). (TIF) [file pone.0166733.s008.tif]

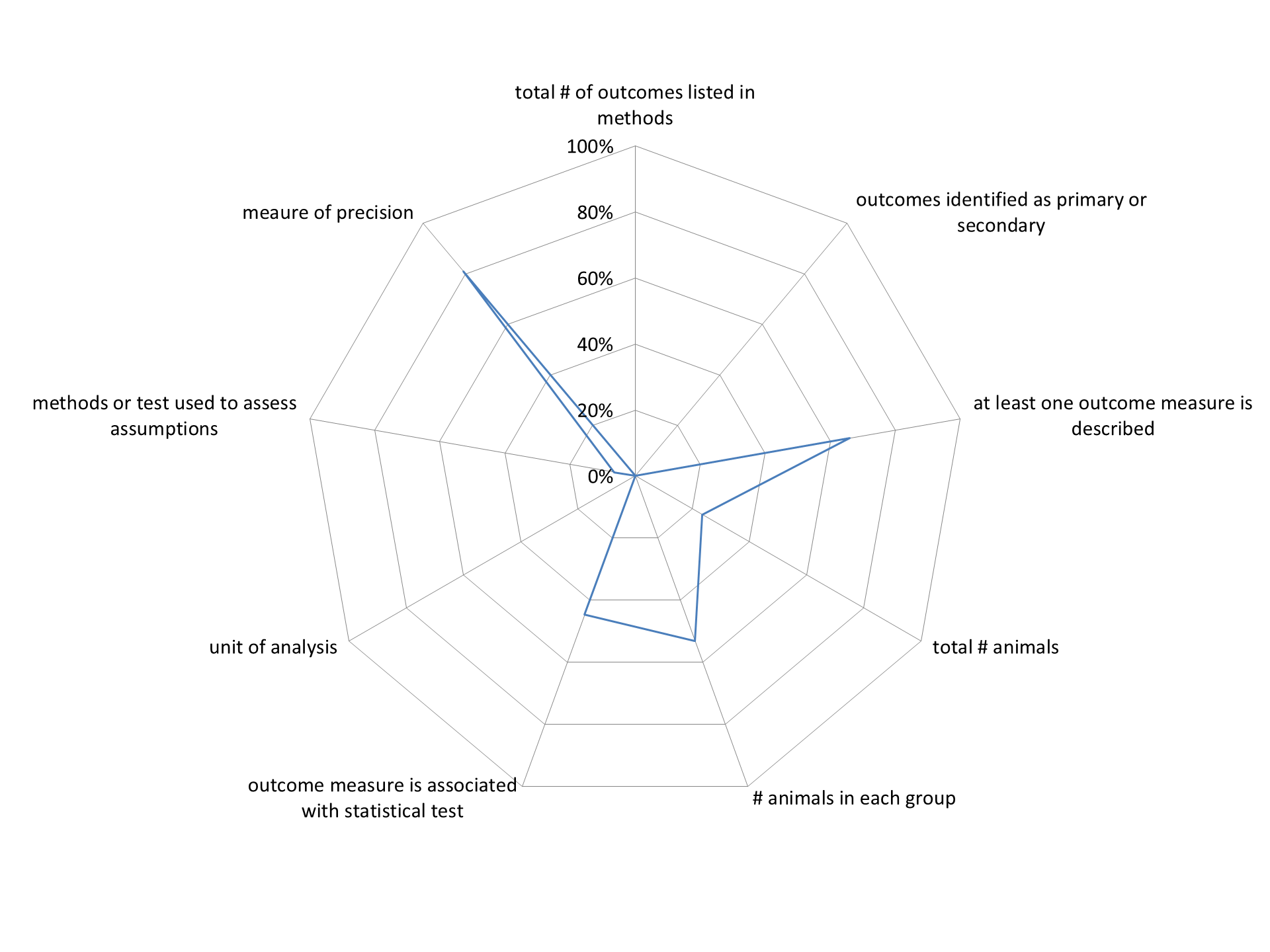

Supplement: S9 Fig — The line represents the percentage of 47 studies that reported the sub-item (e.g. 0% of studies reported the sub-item total # of outcomes listed in methods). (TIF) [file pone.0166733.s009.tif]

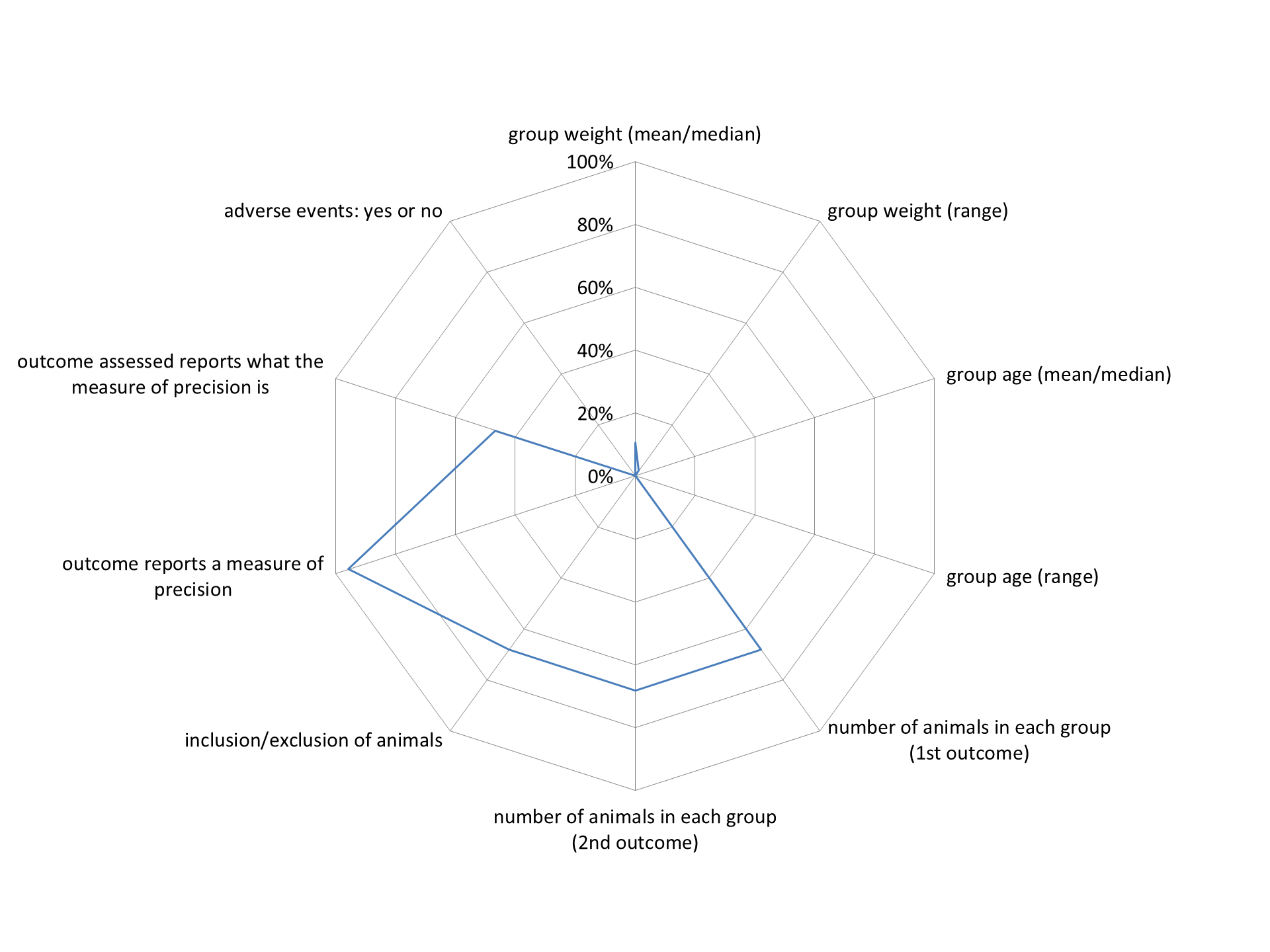

Supplement: S10 Fig — The line represents the percentage of 47 studies that reported the sub-item (e.g. 11% of studies reported the sub-item group weight (mean/median)). (TIF) [file pone.0166733.s010.tif]

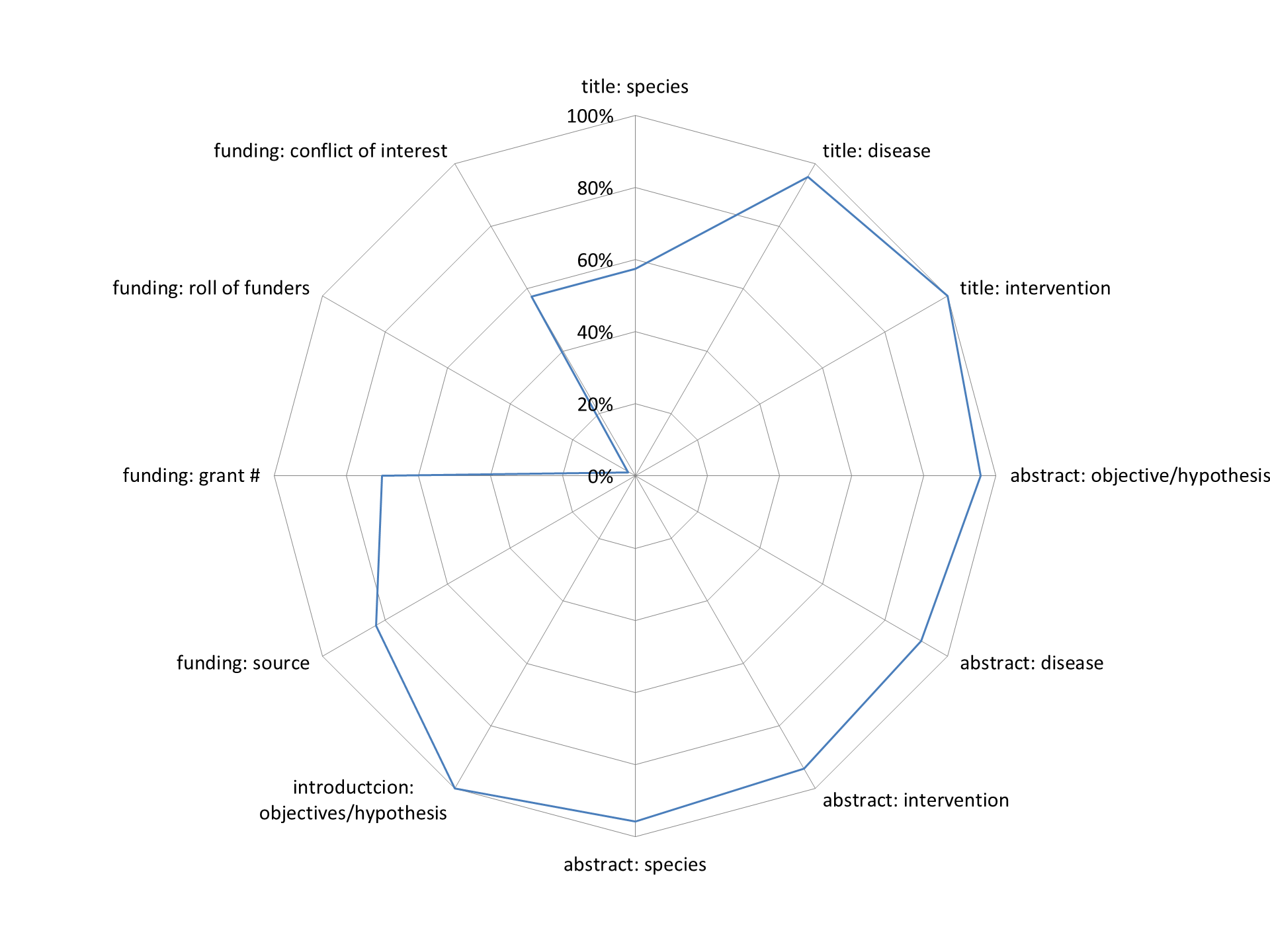

Supplement: S11 Fig — The line represents the percentage of 47 studies that reported the sub-item (e.g. 57% of studies reported the sub-item title: species). (TIF) [file pone.0166733.s011.tif]

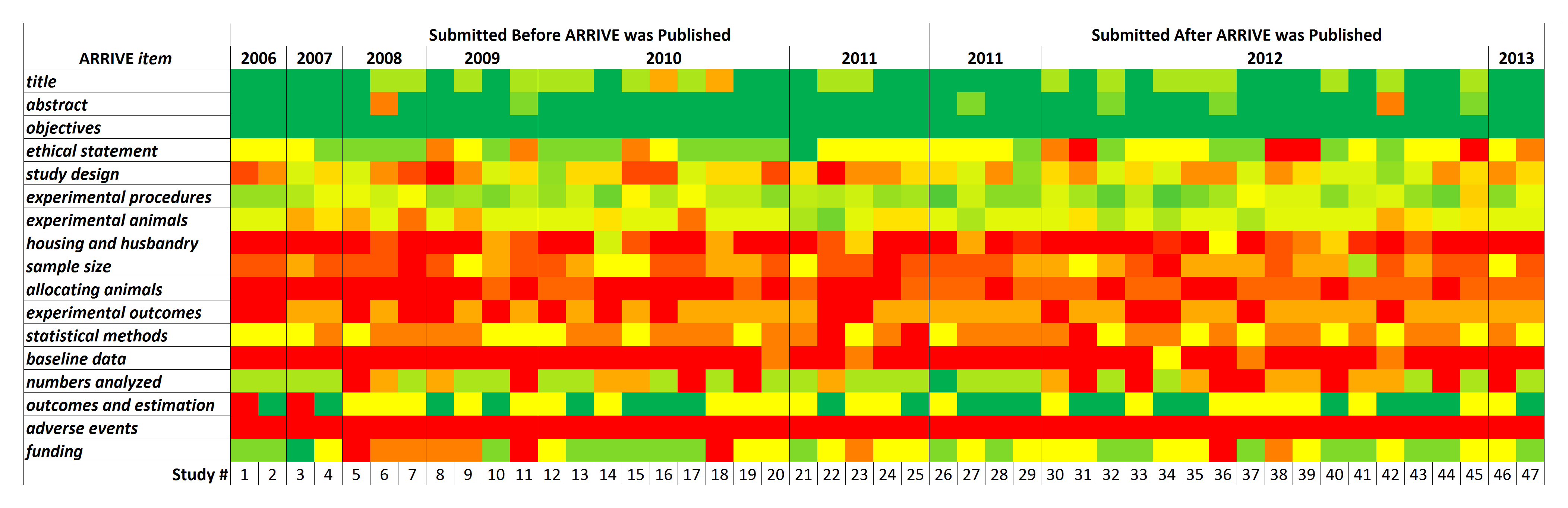

Supplement: S12 Fig — The total number of sub-items for each study was summed by item (e.g. study design) and divided by the total number of sub-items in that item (i.e for title there were three sub-items, thus each study could score: 0[none], 0.33[1 of 3], 0.67[2 of 3], or 1[3 of 3]). Colours were assigned with red = 0 (none reported), yellow = 0.5, green = 1 (all reported). Each column of colour represents one study (e.g. 2006 has two studies), single black lines separate years, and the double black line separates submitted before and after ARRIVE publication. (TIF) [file pone.0166733.s012.tif]

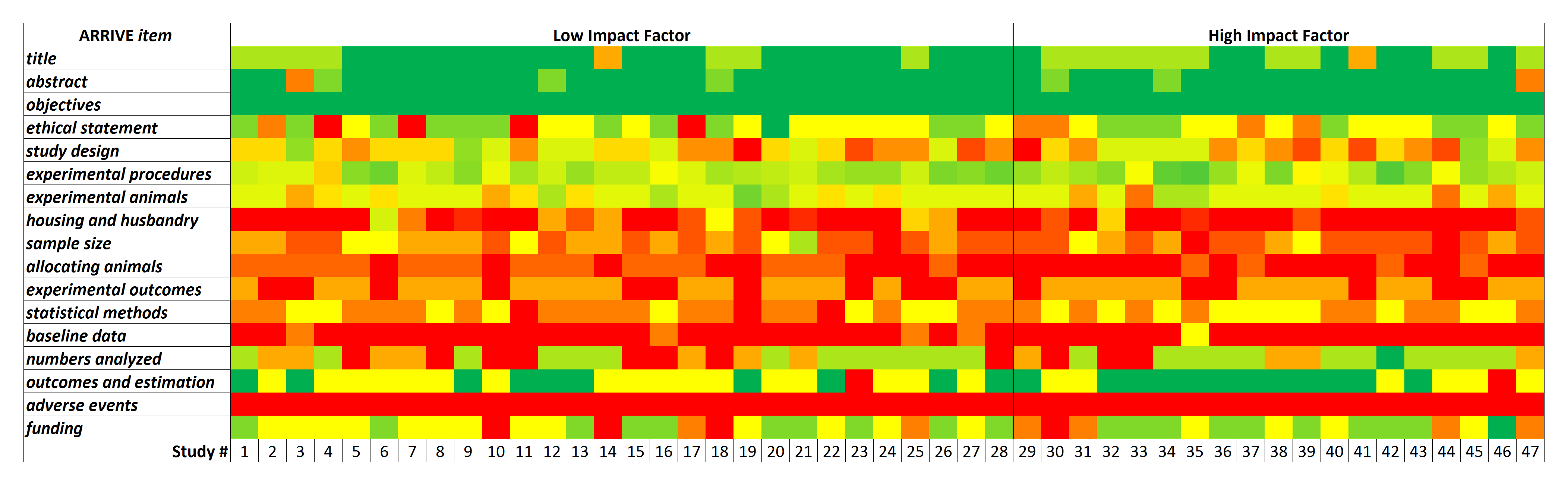

Supplement: S13 Fig — The total number of sub-items for each study was summed by item (e.g. study design) and divided by the total number of sub-items in that item (i.e for title there were three sub-items, thus each study could score: 0[none], 0.33[1 of 3], 0.67[2 of 3], or 1[3 of 3]). Colours were assigned with red = 0 (none reported), yellow = 0.5, green = 1 (all reported). Each column of colour represents one study and the double black line separates low impact factor (<4; n = 28; min = 0, max = 3.62) and high impact factor (> = 4; n = 19; min = 4.02, max = 24.03) study groups. (TIF) [file pone.0166733.s013.tif]
