# Supplementary material for: The Devil Is in the Details: Incomplete Reporting in Preclinical Animal Research
Source: PLoS One. 2016 Nov 17;11(11):e0166733. doi: 10.1371/journal.pone.0166733 (PMC5113978; doi:10.1371/journal.pone.0166733)
Supplement: S1 Table — (PDF) [file pone.0166733.s014.pdf]

Supplementary Table 1. References for Included Studies

1. Mei, S. H. J. *et al.* Prevention of LPS-induced acute lung injury in mice by mesenchymal stem cells overexpressing angiopoietin 1. *PLoS Med.* **4,** e269 (2007).

2. Xu, J. *et al.* Prevention of endotoxin-induced systemic response by bone marrow-derived mesenchymal stem cells in mice. *Am. J. Physiol. Lung Cell. Mol. Physiol.* **293,** L131–41 (2007).

3. Xu, J. *et al.* Mesenchymal stem cell-based angiopoietin-1 gene therapy for acute lung injury induced by lipopolysaccharide in mice. *J. Pathol.* **214,** 472–81 (2008).

4. Zhao, F. *et al.* Therapeutic Effects of Bone Marrow-Derived Mesenchymal Stem Cells Engraftment on Bleomycin-Induced Lung Injury in Rats. *Transplant. Proc.* **40,** 1700–1705 (2008).

5. Gonzalez-Rey, E. *et al.* Human adult stem cells derived from adipose tissue protect against experimental colitis and sepsis. *Gut* **58,** 929–939 (2009).

6. Leblond, A.-L. *et al.* Developing Cell Therapy Techniques for Respiratory Disease: Intratracheal Delivery of Genetically Engineered Stem Cells in a Murine Model of Airway Injury. *Hum. Gene Ther.* **20,** 1329–1343 (2009).

7. Moodley, Y. *et al.* Human umbilical cord mesenchymal stem cells reduce fibrosis of bleomycin-induced lung injury. *Am. J. Pathol.* **175,** 303–313 (2009).

8. Németh, K. *et al.* Bone marrow stromal cells attenuate sepsis via prostaglandin E(2)-dependent reprogramming of host macrophages to increase their interleukin-10 production. *Nat. Med.* **15,** 42–9 (2009).

9. Francisco, S. *et al.* Antibacterial effect of human mesenchymal stem cells is mediated in part from secretion of the antimicrobial peptide LL-37. *Stem Cells* **28,** 2229–2238 (2010).

10. Lee, S.-H. *et al.* Modulation of cytokine and nitric oxide by mesenchymal stem cell transfer in lung injury/fibrosis. *Respir. Res.* **11,** 16 (2010).

11. Liang-kuan, B. I., Bing, T., Bin, Z. H. U., Chun-ling, X. I. E. & Shuang, L. I. Systemic Delivery of IL-10 by Bone Marrow Derived Stromal Cells Has Therapeutic Benefits in Sepsis Therapy *. *Prog. Biochem. Biophys.* **37,** 678–686 (2010).

12. Mei, S. H. J. *et al.* Mesenchymal stem cells reduce inflammation while enhancing bacterial clearance and improving survival in sepsis. *Am. J. Respir. Crit. Care Med.* **182,** 1047–1057 (2010).

13. Yagi, H. *et al.* Bone marrow mesenchymal stromal cells attenuate organ injury induced by LPS and burn. *Cell Transplant.* **19,** 823–830 (2010).

14. Yagi, H. *et al.* Reactive bone marrow stromal cells attenuate systemic inflammation via sTNFR1. *Mol. Ther.* **18,** 1857–1864 (2010).

15. Zhu, F., Guo, G., Chen, W. & Wang, N. Effects of bone marrow-derived mesenchymal stem cells engraftment on vascular endothelial cell growth factor in lung tissue and plasma

at early stage of smoke inhalation injury Preparation of MSCs. *World J. Emerg. Med.* **1,** 224–228 (2010).

16. Danchuk, S. *et al.* Human multipotent stromal cells attenuate lipopolysaccharide-induced acute lung injury in mice via secretion of tumor necrosis factor-α-induced protein 6. *Stem Cell Res. Ther.* **2,** 27 (2011).

17. Hannoush, E. J. *et al.* Impact of Enhanced Mobilization of Bone Marrow Derived Cells to Site of Injury. *J. Trauma Inj. Infect. Crit. Care* **71,** 283–291 (2011).

18. Kim, E. S. *et al.* Intratracheal transplantation of human umbilical cord blood-derived mesenchymal stem cells attenuates Escherichia coli-induced acute lung injury in mice. *Respir. Res.* **12,** 108 (2011).

19. Liang, Z. *et al.* Bone marrow-derived mesenchymal stem cells protect rats from endotoxin-induced acute lung injury. *Chin. Med. J. (Engl).* **124,** 2715–22 (2011).

20. Sun, C. *et al.* Autologous Transplantation of Adipose-Derived Mesenchymal Stem Cells Markedly Reduced Acute Ischemia-Reperfusion Lung Injury in a Rodent Model. *J. Transl. Med.* **9,** 118 (2011).

21. Sun, J., Han, Z., Liao, W. & Yang, S. Intrapulmonary delivery of human of human umbilical cord mesenchymal stem cells attenuates acute lung injury by expanding CD4+ CD25+ Forkhead Boxp3 (FOXP3)+ regulatory T cells and balancing. *Cell. Physiol. …* **300020,** 587–96 (2011).

22. Chang, C.-L. *et al.* Impact of apoptotic adipose-derived mesenchymal stem cells on attenuating organ damage and reducing mortality in rat sepsis syndrome induced by cecal puncture and ligation. *J. Transl. Med.* **10,** 244 (2012).

23. Chen, S. *et al.* Ischemia postconditioning and mesenchymal stem cells engraftment synergistically attenuate ischemia reperfusion-induced lung injury in rats. *J. Surg. Res.* **178,** 81–91 (2012).

24. Chien, M.-H. *et al.* Systemic human orbital fat-derived stem/stromal cell transplantation ameliorates acute inflammation in lipopolysaccharide-induced acute lung injury. *Crit. Care Med.* **40,** 1245–1253 (2012).

25. Curley, G. F. *et al.* Mesenchymal stem cells enhance recovery and repair following ventilator-induced lung injury in the rat. *Thorax* **67,** 496–501 (2012).

26. Gupta, N. *et al.* Mesenchymal stem cells enhance survival and bacterial clearance in murine Escherichia coli pneumonia. *Thorax* **67,** 533–9 (2012).

27. Ionescu, L. *et al.* Stem cell conditioned medium improves acute lung injury in mice: in vivo evidence for stem cell paracrine action. *Am. J. Physiol. Lung Cell. Mol. Physiol.* **303,** L967–77 (2012).

28. Krasnodembskaya, A. *et al.* Human mesenchymal stem cells reduce mortality and bacteremia in gram-negative sepsis in mice in part by enhancing the phagocytic activity of blood monocytes. *Am. J. Physiol. Lung Cell. Mol. Physiol.* **302,** L1003–13 (2012).

29. Li, J., Li, D., Liu, X., Tang, S. & Wei, F. Human umbilical cord mesenchymal stem cells reduce systemic inflammation and attenuate LPS-induced acute lung injury in rats. *J. Inflamm. (Lond).* **9,** 33 (2012).

30. Qin, Z. *et al.* Intrapleural delivery of MSCs attenuates acute lung injury by paracrine/endocrine mechanism. *J. Cell. Mol. Med.* **16,** 2745–53 (2012).

31. Song, L. *et al.* A therapeutic role for mesenchymal stem cells in acute lung injury independent of hypoxia-induced mitogenic factor. *J. Cell. Mol. Med.* **16,** 376–85 (2012).

32. Tai, W. L., Dong, Z. X., Zhang, D. D. & Wang, D. H. Therapeutic effect of intravenous bone marrow-derived mesenchymal stem cell transplantation on early-stage LPS-induced acute lung injury in mice. *Nan Fang Yi Ke Da Xue Xue Bao* **32,** 283–290 (2012).

33. Wang, L., Tu, X.-H., Zhao, P., Song, J.-X. & Zou, Z.-D. Protective effect of transplanted bone marrow-derived mesenchymal stem cells on pancreatitis-associated lung injury in rats. *Mol. Med. Rep.* **6,** 287–92 (2012).

34. Wu, Q. *et al.* The effect of allogenetic bone marrow-derived mesenchymal stem cell transplantation on lung aquaporin-1 and -5 in a rat model of severe acute pancreatitis. *Hepatogastroenterology.* **59,** 965–976 (2012).

35. Xu, Y. *et al.* Intravenous transplantation of mesenchymal stem cells attenuates oleic acid induced acute lung injury in rats. *Chin. Med. J. (Engl).* **125,** 2012–2018 (2012).

36. Curley, G. F. *et al.* Effects of intratracheal mesenchymal stromal cell therapy during recovery and resolution after ventilator-induced lung injury. *Anesthesiology* **118,** 924–32 (2013).

37. Hannoush, E. J. *et al.* Role of bone marrow and mesenchymal stem cells in healing after traumatic injury. *Surgery* **153,** 44–51 (2013).

38. Lim, R. *et al.* Human Mesenchymal Stem Cells Reduce Lung Injury in Immunocompromised Mice but Not in Immunocompetent Mice. *Respiration* **85,** 332–341 (2013).

39. Maron-Gutierrez, T. *et al.* Effects of mesenchymal stem cell therapy on the time course of pulmonary remodeling depend on the etiology of lung injury in mice. *Crit. Care Med.* **41,** e319–33 (2013).

40. Shin, S. *et al.* The therapeutic effect of human adult stem cells derived from adipose tissue in endotoxemic rat model. *Int. J. Med. Sci.* **10,** 8–18 (2013).

41. Wen, Q. Adipose tissue-derived stem cells attenuate acute lung injury through eNOS and eNOS-derived NO. *Int. J. Mol. Med.* 1313–1318 (2013). doi:10.3892/ijmm.2013.1328

42. Yang, B. *et al.* Effect of umbilical cord mesenchymal stem cells on treatment of severe acute pancreatitis in rats. *Cytotherapy* **15,** 154–62 (2013).

43. Yang, H. *et al.* Combined treatment with bone marrow mesenchymal stem cells and methylprednisolone in paraquat-induced acute lung injury. *BMC Emerg. Med.* **13 Suppl 1,** S5 (2013).

44. Yilmaz, S. *et al.* Mesenchymal stem cell: does it work in an experimental model with acute respiratory distress syndrome? *Stem Cell Rev.* **9,** 80–92 (2013).

45. Zhang, S. *et al.* Comparison of the therapeutic effects of human and mouse adipose-derived stem cells in a murine model of lipopolysaccharide-induced acute lung injury. *Stem Cell Res. Ther.* **4,** 13 (2013).

46. Zhao, Y. *et al.* Therapeutic effects of bone marrow-derived mesenchymal stem cells on pulmonary impact injury complicated with endotoxemia in rats. *Int. Immunopharmacol.* **15,** 246–253 (2013).
