# Supplementary material for: The Devil Is in the Details: Incomplete Reporting in Preclinical Animal Research
Source: PLoS One. 2016 Nov 17;11(11):e0166733. doi: 10.1371/journal.pone.0166733 (PMC5113978; doi:10.1371/journal.pone.0166733)
Supplement: S2 Table — (PDF) [file pone.0166733.s015.pdf]

Supplementary Table 2. Operationalized Reporting sub-items from the ARRIVE Guidelines and Examples

ARRIVE Section	ARRIVE # and *item*	sub-items	Examples
Title	*1 title*	1.1 species studied<br>1.2 disease modeled<br>1.3 intervention tested	1.1 Mice, or Murine etc.<br>1.2 Acute lung injury or sepsis etc.<br>1.3 MSCs etc.
Abstract	*2 abstract*	2.1 the objective or hypothesis<br>2.2 disease model is stated in the objective or hypothesis<br>2.3 intervention is stated in the objective or hypothesis<br>2.4 the species or strain studied is stated anywhere in the abstract	2.1 Any objective or hypothesis<br>2.2 e.g. acute lung injury; but must be included in objective/hypothesis<br>2.3 e.g. MSCs; but must be included in objective/hypothesis<br>2.4 e.g. Mice or murine; can be located anywhere in abstract
Introduction	*3 background*	removed	
	4 objectives	4.1 the objective or hypothesis is stated	4.1 Any statement indicating the objective/hypothesis will suffice
Methods	*5 ethical statement*	5.1 explicit statement of approval<br>5.2 approval body name<br>5.3 name of international, national or	5.1 e.g. “All experimental animal procedures were approved by the Institute of Animal Care and Use Committee…”

	institutional guidelines followed<br>5.4 list an ethics protocol/permit number	5.2 e.g. "… by the Institute of Animal Care and Use Committee at Kaohsiung Chang Gung Memorial Hospital<br>5.3 e.g. "…and performed in accordance with the Guide for the Care and Use of Laboratory Animals." Can be any guidelines and can be listed by name or more generally such as 'institutional guidelines'.<br>5.4 *e.g. "*Affidavit of Approval of Animal Use Protocol No. 2008121108"
6 study design	6.1 the total number of experimental and control groups is listed<br>6.2. the # of *in vivo* experimental and control groups assigned to a receive treatment is internally consistent within the methods<br>6.3 the # of *in vivo* experimental and control groups assigned to receive treatment is consistent between the methods and results	6.1 e.g. "Rats were randomly assigned to one of three experimental groups".<br>6.2 e.g. "Rats were randomly assigned to one of three experimental groups 1) saline solution plus saline treatment (n=5), 2) LPS plus saline treatment (n=5), and 3) LPS plus hATSCs treatment (n=5)."<br>6.3 If there are three groups (as in example 6.1) there must be 3, and only 3 groups, in the results section.<br>6.4 e.g. "In the study, n refers to number of animals…"<br>6.5 Personnel are reported as blinded for at least one task,

	6.4 the experimental unit<br>6.5 blinding of personnel<br>6.6 blinding of outcome assessment<br>6.7 diagram of experimental design	or why blinding is not conducted is reported<br>6.6 An assessor is blinded for at least one of the outcomes measured, or why blinding is not conducted is reported<br>6.7 Any diagram for any aspect of the experimental design that includes assigning animals treatment
7 experimental procedures (model)	7.1 drug<br>7.2 drug vehicle<br>7.3 drug vehicle Volume<br>7.4 drug dose<br>7.5 route<br>7.6 site<br>7.7 supplier<br>7.8 when<br>7.9 where<br>7.10 was anesthesia use reported<br>7.11 anesthesia (route)<br>7.12 anesthesia (type)<br>7.13 anesthesia (dose)	7.1 (e.g. Lipopolysaccharide)<br>7.2 (e.g. PBS)<br>7.3 (e.g. 100 µL)<br>7.4 (e.g. 10mg/kg)<br>7.5 (e.g. intravenous)<br>7.6 (e.g. tail vein)<br>7.7 (e.g. Sigma Aldrich)<br>7.8 (e.g. “Except the burrowing assay, which was conducted from the beginning of the dark cycle, all other behavioural experiments were conducted in the light phase”)<br>7.9 (e.g. home cage, laboratory, etc.)<br>7.10 (e.g. Rats were anesthetized…)

		7.14 was analgesia use reported	7.11 (e.g. intravenous)<br>7.12 (e.g. isoflurane)<br>7.13 (e.g. xx mg/kg)<br>7.14 (e.g. We used analgesia…)
	7 experimental procedures (MSCs)	7.1 MSC species source	7.1 (e.g. Xenogenic Human)
		7.2 MSC species source sex	7.2 (e.g. Male)
		7.3 MSC tissues type	7.3 (e.g. Adipose, Bone Marrow, etc.)
		7.4 MSC source supplier	7.4 (e.g. State Stem Cell Industry Base)
		7.5 MSC vehicle	7.5 (e.g. PBS)
		7.6 MSC vehicle volume	7.6 (e.g. MSC per/mL)
		7.7 MSC dose	7.7 (e.g. 50,000/mL)
		7.8 MSC route	7.8 (e.g. intravenous)
		7.9 MSC site	7.9 (e.g. tail vein)
		7.10 MSC frequency of administration	7.10 (Once, Twice etc.)
		7.11 MSC when	7.11 (e.g. time after model inducement)
		7.12 MSC where	7.12 (e.g. home cage, laboratory, etc.)
		7.13 MSC rationale for drug dose or timing of dose	7.13 (any explanation will do)

	7 experimental procedures (controls)	7.1 control drug	7.1 (e.g. Normal Saline)
		7.2 control dose	7.2 (e.g. 100mL or 1mL/kg)
		7.3 control route of administration (type)	7.3 (e.g. Intravenous)
		7.4 control site of administration	7.4 (e.g. Tail Vein)
		7.5 control frequency of administration	7.5 (e.g. Once, Twice etc.)
		7.6 control when	7.6 (e.g. time after model inducement)
		7.7 control where	7.7 (e.g. home cage, laboratory, etc.)
	7 experimental procedures (euthanasia)	7.1 euthanasia reported	7.1 (do the authors indicate that the animals were sacrificed/euthanized etc.?)
		7.2 euthanasia method	7.2 (e.g. exsanguination)
		7.3 analgesia use reported	7.3 (e.g. We used analgesia…)
	8 experimental animals	8.1 animal species	8.1 (Latin or common name)
		8.2 strain	8.2 (international strain nomenclature)
		8.3 sex	8.3 (male or female)
		8.4 age	8.4 (all animals: mean or median)
		8.5 age	8.5 (all animals: range)
		8.6 weight	8.6 (all animals: mean or median)
		8.7 weight	8.7 (all animals: range)

	8.8 source	8.8 (supplier)
	8.9 health immune status	8.9 (state that animals are SPF or SCID etc.)
9 housing and husbandry	9.1 type of facility	9.1 (e.g. specific pathogen free)
	9.2 type of cage or housing	9.2 (e.g. Rack type, cage dimensions etc.)
	9.3 bedding material	9.3 (e.g. type, supplier etc.)
	9.4 cage companions	9.4 (e.g. housed individual or group)
	9.5 light/dark cycle	9.5 (e.g. 12h light/dark)
	9.6 temperature	9.6 (e.g. 23 degree C average)
	9.7 quality of water	9.7 (e.g. tap water or distilled water etc.)
	9.8 type of food	9.8 (e.g. any food supplier name)
	9.9 access to food	9.9 (e.g. ad libitum)
	9.10 access to water	9.10 (e.g. ad libitum)
	9.11 environmental enrichment	9.11 (e.g. any environmental enrichment)
	9.12 welfare assessment/intervention before/during/after experiment	9.12 (e.g. any welfare assessment at any time)
10 sample size	10.1 total number of animal used, no addition required) 10.2 number of animals in each	10.1 (e.g. “In total 120 mice were used”; must state the total number of animals for all in vivo experiments, make sure the authors don’t add additional groups/experiments)

		experimental group<br>10.3 was sample-size calculation conducted<br>10.4 statistical method for the sample-size calculation reported or any other explanation provided<br>10.5 indicates if experiment was repeated<br>10.6 indicates biological or technical replicates	10.2 (the exact number, not range for all groups including sub-groups)<br>10.3 State that sample size calculation was conducted<br>10.4 Statistical method or explanation of how sample size was determined<br>10.5 All experiments were repeated at least twice<br>10.6 Three lung sections from each rat were analyzed and three randomly selected high-power fields (HPFs) (100×) were examined in each section
	11 allocating animals to experimental groups	11.1 animals were randomized to groups<br>11.2 random sequence generation described<br>11.3 allocation concealment described<br>11.4 describes order in which animals in different experimental groups were treated<br>11.5 describes order in which animals in different experimental groups were	11.1 (must state that animals were randomized for to at least one set of groups, but does not need to state randomization for all groups).<br>11.2 (Description of how random sequence was generated)<br>11.3 (Description of how allocation was concealed)<br>11.4 (any description about the order in which animals were treated)<br>11.5 (any description about the order in which animals assessed)

	assessed	
12 experimental outcomes	12.1 the total number of outcomes is listed in the methods<br>12.2 outcomes are identified as being either primary or secondary<br>12.3 at least one outcome measure listed is described	12.1 (e.g. “Two primary outcomes and three secondary outcomes were analyzed”.)<br>12.2 (e.g. “Two primary outcomes and three secondary outcomes were analyzed”)<br>12.3 (e.g. “Two primary outcome measures were analyzed: overall performance on the MWM (days 12-16) and the numbers of surviving CA2-3 cells…
13 statistical methods	13.1 at least one outcome measure is associated with at least one statistical test<br>13.2 unit of analysis for at least one tests<br>13.3 describes method or states test used to assess assumptions for statistical approach(es)<br>13.4 at least one measure of precision for at least one analysis.	13.1 The statistical analysis of cells expressing proinflammatory cytokines was performed using paired t-test.<br>13.2 For each test, the experimental unit was an individual animal.<br>13.3 Test for normality was performed by Kolmogorov–Smirnov test. Must state methods or test, not just that data was normal.<br>13.4 Standard Deviation, Standard Error, Confidence Intervals etc. and which analyses they apply to is reported.

Results	*14 baseline data*	14.1 weight for each group<br>14.2 weight for each group<br>14.3 age for each group<br>14.4 age for each group	14.1 (mean or median)<br>14.2 (range)<br>14.3 (mean or median)<br>14.4 (range)
	15 numbers analysed	15.1 number of animals in each group for mortality. If mortality is not measure than the first outcome measure reported.<br>15.2 number of animals in each group for either the first outcome after mortality or if no mortality than the second outcome.<br>15.3 inclusion/exclusion of animals (for any outcome)	15.1 (absolute numbers for each in each analysis, not % or range)<br>15.2 (absolute numbers for each in each analysis, not % or range)<br>15.3 Any statement indicating that either all animals/data were included or that some were excluded for any outcome
	16 outcomes and estimation	16.1 either of the outcomes from 15.1 or 15.2 must report a measure of precision.<br>16.2 either of the outcomes from 15.1 or 15.2 must state what the measure of precision is.	16.1 Standard Deviation, Standard Error of the Mean, Confidence Interval; can be displayed graphically.<br>16.2 Must state what measure of precision is in text or graphic e.g. "error bar are SEM".
	17 adverse events	17.1 a statement indicating that adverse	17.1 (e.g. "In four surviving animals, lower extremity

		events occurred or did not occur for at least one experimental group.	ulcers developed but were effectively treated with local standard triple antibiotic ointment (bacitracin, neomycin, and Polymyxin B) and cohesive bandages”. Adverse Event: Any event that is reported (e.g. diarrhea) that is reported but not an outcome measure)
Discussion	*18 interpretation/scientific implications*	removed	
	19 generalisability/translation	removed	
	20 funding	20.1 funding source(s) declared (funding source)<br>20.2 grant etc. must include number (grant #)<br>20.3 roll of funders described<br>20.4 statement of competing/conflict of interest	20.1 e.g. This work was supported by a grant from CIHR.<br>20.2 e.g. This work was supported by a grant from CIHR (#12345).<br>20.3 e.g. The funders had no role in study design, data collection and analysis, decision to publish, or preparation of the manuscript.<br>20.4 e.g. the authors declare no conflicts of interest
