# Supplementary material for: The Devil Is in the Details: Incomplete Reporting in Preclinical Animal Research
Source: PLoS One. 2016 Nov 17;11(11):e0166733. doi: 10.1371/journal.pone.0166733 (PMC5113978; doi:10.1371/journal.pone.0166733)
Supplement: S3 Table — (PDF) [file pone.0166733.s016.pdf]

Supplementary Table 3. Number and Percentage of Studies Reporting for Each sub-item

ARRIVE Section	ARRIVE *item*	sub-item #	# of Studies Reporting	% of Studies Reporting
**Title**	***title***	1.1	27	57%
		1.2	45	96%
		1.3	47	100%
**Title Summary**		**3**	**119**	**84%**
**Abstract**	***abstract***	2.1	45	96%
		2.2	43	91%
		2.3	44	94%
		2.4	45	96%
**Abstract Summary**		**4**	**177**	**94%**
**Introduction**	***objectives***	4.1	47	100%
**Introduction Summary**		**1**	**47**	**100%**
**Methods**	***ethical statement***	5.1	39	83%
		5.2	37	79%
		5.3	24	51%
		5.4	1	2%
	study design	6.1	15	32%
		6.2	38	81%
		6.3	33	70%
		6.4	4	9%
		6.5	2	4%
		6.6	27	57%
		6.7	11	23%
	experimental procedures	7.1 Model	47	100%

7.2 Model	25	53%
7.3 Model	25	53%
7.4 Model	40	85%
7.5 Model	47	100%
7.6 Model	43	91%
7.7 Model	35	74%
7.8 Model	0	0%
7.9 Model	0	0%
7.10 Model	33	70%
7.11 Model	24	51%
7.12 Model	29	62%
7.13 Model	23	49%
7.14 Model	0	0%
7.1 MSC	45	96%
7.2 MSC	19	40%
7.3 MSC	43	91%
7.4 MSC	45	96%
7.5 MSC	31	66%
7.6 MSC	33	70%
7.7 MSC	46	98%
7.8 MSC	46	98%
7.9 MSC	42	89%
7.10 MSC	47	100%
7.11 MSC	44	94%
7.12 MSC	0	0%

	7.13 MSC	4	9%
	7.1 Control	45	96%
	7.2 Control	32	68%
	7.3 Control	45	96%
	7.4 Control	40	85%
	7.5 Control	44	94%
	7.6 Control	40	85%
	7.7 Control	0	0%
	7.1 Euthanasia	38	81%
	7.2 Euthanasia	16	34%
	7.3 Euthanasia	12	26%
experimental animals	8.1	47	100%
	8.2	45	96%
	8.3	37	79%
	8.4	2	4%
	8.5	21	45%
	8.6	3	6%
	8.7	25	53%
	8.8	35	74%
	8.9	6	13%
housing and husbandry	9.1	7	15%
	9.2	2	4%
	9.3	0	0%
	9.4	1	2%
	9.5	7	15%

		9.6	5	11%
		9.7	2	4%
		9.8	5	11%
		9.9	12	26%
		9.10	12	26%
		9.11	0	0%
		9.12	0	0%
	sample size	10.1	11	23%
		10.2	25	53%
		10.3	1	2%
		10.4	1	2%
		10.5	8	17%
		10.6	27	57%
	allocating animals to	11.1	22	47%
	experimental groups	11.2	0	0%
		11.3	0	0%
		11.4	0	0%
		11.5	0	0%
	experimental outcomes	12.1	0	0%
		12.2	0	0%
		12.3	31	66%
	statistical methods	13.1	21	45%
		13.2	0	0%
		13.3	3	6%
		13.4	38	81%

**Methods Summary**		**87**	**1821**	**45%**
**Results**	***baseline data***	14.1	5	11%
		14.2	1	2%
		14.3	0	0%
		14.4	0	0%
	numbers analyzed	15.1	32	68%
		15.2	28	60%
		15.3	2	4%
	outcomes and estimation	16.1	45	96%
		16.2	22	47%
	adverse events	17.1	0	0%
**Results Summary**		**10**	**135**	**29%**
**Discussion**	***funding***	20.1	39	83%
		20.2	33	70%
		20.3	1	2%
		20.4	27	57%
**Discussion Summary**		**4**	**100**	**53%**
