# Supplementary material for: The Devil Is in the Details: Incomplete Reporting in Preclinical Animal Research
Source: PLoS One. 2016 Nov 17;11(11):e0166733. doi: 10.1371/journal.pone.0166733 (PMC5113978; doi:10.1371/journal.pone.0166733)
Supplement: S4 Table — (PDF) [file pone.0166733.s017.pdf]

Supplementary Table 4. NIH Core Reporting *item* and Matched ARRIVE sub-items

NIH *item*	ARRIVE sub-items	Total # (%) of Studies
replicates	10.5 indicates the experiment was repeated	8 (17%)
	10.6 indicates repeating of biological or technical replicates	27 (57%)
statistics	13.1 at least one outcome measure is associated with at least one statistical test	21 (77%)
	13.2 unit of analysis for at least one test	0 (0%)
	13.3 describes method or test used to assess assumptions for statistical approach(es)	3 (6%)
	13.4 at least one measure of precision for at least one analysis	38 (81%)
randomization	11.1 animals were randomized to groups	22 (47%)
	11.2 random sequence generation described	0 (0%)
blinding	6.5 blinding of personnel	2 (4%)
	6.6 blinding of outcome assessment	27 (57%)
sample-size estimation	10.3 was a sample-size calculation conducted	1 (2%)
	10.4 statistical method for the sample-size calculation reported or any other explanation provided	1 (2%)
inclusion and exclusion criteria	15.3 inclusion/exclusion of animals (for any outcome)	2 (4%)
